# Ablation of *Iah1*, a candidate gene for diet-induced fatty liver, does not affect liver lipid accumulation in mice

**DOI:** 10.1371/journal.pone.0233087

**Published:** 2020-05-14

**Authors:** Tomomi Masuya, Miyako Suzuki, Junko Tsujimura, Shinsaku Kanamori, Yuki Miyasaka, Tamio Ohno, Atsushi Murai, Fumihiko Horio, Misato Kobayashi

**Affiliations:** 1 Laboratory of Animal Nutrition, Department of Animal Sciences, Graduate School of Bioagricultural Sciences, Nagoya University, Nagoya, Japan; 2 Division of Experimental Animals, Center for Promotion of Medical Research and Education, Graduate School of Medicine, Nagoya University, Nagoya, Japan; INRA, FRANCE

## Abstract

Nonalcoholic fatty liver disease (NAFLD) is a pathological condition caused by excess triglyceride deposition in the liver. The SMXA-5 severe fatty liver mouse model has been established from the SM/J and A/J strains. To explore the genetic factors involved in fatty liver development in SMXA-5 mice, we had previously performed quantitative trait locus (QTL) analysis, using (SM/J×SMXA-5)F2 intercross mice, and identified *Fl1sa* on chromosome 12 (centromere-53.06 Mb) as a significant QTL for fatty liver. Furthermore, isoamyl acetate-hydrolyzing esterase 1 homolog (*Iah1*) was selected as the most likely candidate gene for *Fl1sa*. *Iah1* gene expression in fatty liver-resistant A/J-12^SM^ mice was significantly higher than in fatty liver-susceptible A/J mice. These data indicated that the *Iah1* gene might be associated with fatty liver development. However, the function of murine *Iah1* remains unknown. Therefore, in this study, we created *Iah1* knockout (KO) mice with two different backgrounds [C57BL/6N (B6) and A/J-12^SM^ (A12)] to investigate the relationship between *Iah1* and liver lipid accumulation. Liver triglyceride accumulation in *Iah1*-KO mice of B6 or A12 background did not differ from their respective *Iah1*-wild type mice under a high-fat diet. These results indicated that loss of *Iah1* did not contribute to fatty liver. On the other hands, adipose tissue dysfunction causes lipid accumulation in ectopic tissues (liver, skeletal muscle, and pancreas). To investigate the effect of *Iah1* deficiency on white adipose tissue, we performed DNA microarray analysis of epididymal fat in *Iah1*-KO mice of A12 background. This result showed that *Iah1* deficiency might decrease adipokines *Sfrp4* and *Metrnl* gene expression in epididymal fat. This study demonstrated that *Iah1* deficiency did not cause liver lipid accumulation and that *Iah1* was not a suitable candidate gene for *Fl1sa*.

## Introduction

Nonalcoholic fatty liver disease (NAFLD) refers to a wide range of liver conditions which occur without excessive alcohol consumption, from simple steatosis to steatohepatitis, fibrosis, and cirrhosis [1]. The incidence of NAFLD has been increasing worldwide along with the increased prevalence of obesity, type 2 diabetes, and dyslipidemia [2]. Hepatic steatosis is caused by excessive energy intake and lack of exercise, and occurs when the balance of the lipid metabolism in the liver is disrupted. However, as not only environmental factors, such as diet and exercise, but also genetic factors, affect NAFLD development, the pathogenesis of this disease has not been fully understood.

The SMXA-5 mouse strain is one of the SMXA recombinant inbred strains created from SM/J and A/J mice that are resistant to fatty liver [3]. The SMXA-5 mouse, whose chromosome consists of parental chromosomal fragments, shows severe fatty liver under a short-term high-fat diet [4]. This indicates that a combination of multiple genes causes fatty liver in SMXA-5 mice. To identify the genetic factors involved in fatty liver development in SMXA-5 mice, we previously performed quantitative trait locus (QTL) analysis using (SM/J×SMXA-5)F2 intercross mice [5]. QTLs for liver weight, liver TG content, and total liver lipid content were mapped to the region from the centromere to 53.06 Mb on the mouse chromosome 12 and named *Fl1sa* (Fatty Liver 1 in SMXA) [5]. On *Fl1sa*, the fatty liver-susceptible allele was A/J and the resistant allele was SM/J. The effect of *Fl1sa* was confirmed using A/J-12^SM^ consomic mice, a strain which possesses the resistant chromosome 12 of SM/J mice and the genetic background of A/J mice. DNA microarray analysis in the liver showed that *Iah1* (isoamyl acetate-hydrolyzing esterase 1 homolog) gene expression in fatty liver-resistant A/J-12^SM^ mice was higher than in fatty liver-susceptible A/J mice [6]. We also confirmed by congenic mice analysis that the chromosomal region possessing the *Iah1* gene contained genes responsible for fatty liver development [7]. Therefore, we have selected *Iah1*, which is located at 21.31 Mb on mouse chromosome 12, as the most likely candidate gene for *Fl1sa*. Previous studies have reported that the yeast IAH1 protein has esterase activity [8, 9]. However, the function of murine IAH1 is largely unknown. We previously reported that *Iah1* overexpression in a mouse hepatoma cell line (Hepa1-6) affected gene expression of the lipid metabolism [6].

In this study, to investigate the relationship between *Iah1* and liver lipid accumulation, we produced *Iah1* knockout (KO) mice with a C57BL/6N and A/J-12^SM^ background. We also performed DNA microarray analysis using the epididymal fat tissue of A/J-12^SM^ (WT_A12) and A/J-12^SM^ *Iah1*-KO mice (KO_A12) to investigate the effect of *Iah1* deficiency on lipid metabolism.

## Materials and methods

### Animals

The tm1a (*Iah1*^*tm1a (EUCOMM) Hmgu*^) mice were obtained from the European Conditional Mouse Mutagenesis Program (EUCOMM). The CAG-Cre [B6.Cg-Tg (CAG-Cre) CZ-MO2Osb] [10] mice were obtained from RIKEN BRC (Japan, RBRC01828). The tm1a mice had a C57BL/6NTac genetic background and had a heterozygous knockout-first allele with the loxP and Frt sites (http://www.mousephenotype.org/about-ikmc/eucomm) (S1 Fig) [11]. The CAG-Cre transgenic mice (C57BL/6NCrSlc genetic background) constitutively express the Cre recombinase gene under the control of a CAG promoter. To generate KO mice, the tm1a heterozygotes were crossed with CAG-Cre mice resulting in tm1b heterozygotes which were heterozygous for Cre (mate A in S1 Fig). The genomic construct in tm1b mice had exons 3 and 4 deleted, which leads to the production of a truncated mRNA, thereby resulting in non-sense mediated decay of the RNA. To remove the Cre recombinase, the tm1b heterozygotes (*Iah1*^*tm1b/WT*^, *Cre/WT*) were crossed with mice which had the same genotype and the heterozygotes (*Iah1*^*tm1b/WT*^, *WT/WT*) without Cre were chosen (mate B in S1 Fig). The *Iah1*^*tm1b*^ heterozygotes were intercrossed to generate *Iah1*^*WT/WT*^ (wild type, WT_B6), *Iah1*^*tm1b/WT*^ (heterozygous KO, Hetero), and *Iah1*^*tm1b/tm1b*^ (homozygous KO, KO_B6) (mate C in S1 Fig). These lines were maintained by (heterozygous×heterozygous) breeding. To ameliorate animal suffering, the tails of the mice were collected under anesthesia using isoflurane in order to perform genotyping. DNA extraction from the tail (about 10 mg) was performed using the DNeasy Blood & Tissue kit (QIAGEN). The primer sequences used for genotyping are described in S1 Table.

The A/J-Chr12^SM^ consomic strain (A/J-12^SM^) was obtained from the Institute for Laboratory Animal Research, Nagoya University School of Medicine. The A/J-12^SM^ consomic mouse strain was established by the introduction of chromosome 12 of the SM/J mouse into the A/J mouse background as previously described [12]. Targeted disruption of the *Iah1* gene on the A/J-12^SM^ background was carried out by using the CRISPR/Cas9 method as previously described [13, 14]. The CRISPR RNA (crRNA) sequences were 5ʹ-CTGTGCGAGCGAGCTGCTAG(CGG)-3ʹ (*Iah1*_g21316438-21316460) and 5ʹ-CTTCGGGGACTCCATCACGC(AGG)-3ʹ (*Iah1*_g21316491-21316513). The two crRNAs were designed using the CRISPOR website (http://crispor.tefor.net/) [15]. Founder mice were mated with A/J-12^SM^ mice to produce offspring with heterozygous mutations in the *Iah1* gene. The DNA sequence in the Cas-9 nuclease-target region was confirmed by Sanger sequencing. The primer sequences used were as follows, 5ʹ-GAAGTCAGGCGGTCTACAGTGAG-3ʹ (Forward) and 5ʹ-GGAAGAGAGGAGTGAACTAGTCGG-3ʹ (Reverse). Off-target sites were predicted using CRISPOR [15]. The top four or five highest-scoring genes as ranked by the MIT off-target score were amplified by PCR and subjected to DNA sequence analysis (S2 Table). Finally, to generate A/J-12^SM^ *Iah1* homozygous KO mice (KO_A12), the heterozygous mice were intercrossed. The genomic DNA was amplified by PCR using primers for *Iah1* genotyping, namely 5ʹ-TTTCTACCATGTCGCTGTGC-3ʹ (forward) and 5ʹ-AGGCGGACCCTTTAAGCTC-3ʹ (reverse). The size differences between the PCR products obtained from the *Iah1*-wild type allele and mutation allele were detected by agarose gel electrophoresis. The established KO_A12 line was maintained by (homozygous × homozygous) breeding. A/J-12^SM^ (WT_A12) mice were used as a control for KO_A12 mice.

All mice were maintained at 23 ± 2 °C with a 12-h light/dark cycle (light on from 8:00 to 20:00) and *ad libitum* access to food and water under conventional conditions, in the facilities at the Graduate School of Bioagricultural Sciences, Nagoya University. Mice were weaned at 3 weeks of age and they were housed individual cages at 5 weeks of age.

### Diet and experimental schedule

Male *Iah1*-wild type (WT_B6 or WT_A12) and -KO (KO_B6 or KO_A12) mice were fed with a standard chow, CE-2 (CLEA Japan, Inc., Japan), until 6 weeks of age. Subsequently, all mice were fed with a high-fat diet (D07053003; Research Diets, New Brunswick, NJ, USA) from 6 to 18 weeks of age (for 12 weeks). The composition of the high-fat diet was previously described [7]. Briefly, the high-fat diet used includes lard (30% w/w) and casein (20.9% w/w). The body weight was measured every week during the experimental period (6–18 weeks of age). At 18 weeks of age, blood samples were collected from orbital veins after a 4 h fast (9:00 to 13:00). Then, the mice were sacrificed by cervical dislocation. The tissues (liver, kidney, lung, brown adipose tissue, epididymal fat, subcutaneous fat, mesenteric fat, and retroperitoneal fat) were collected, weighed, and immediately frozen using liquid nitrogen.

This study was carried out in accordance with the Regulations on Animal Experiments of Nagoya University. All procedures and animal care were approved by the Animal Experiment Committee, Graduate School of Bioagricultural Sciences, Nagoya University (approval No. 201622604, 2017030219, 2018031316).

### DNA sequence analysis

The *Iah1* gene and off-target candidate genes of KO_A12 mice were amplified by PCR and the PCR products were purified using the ExoSAP-IT kit (Affymetrix). The primers used for sequence analysis of off-target effects are shown in S2 Table. The purified PCR products were labeled using the BigDye Terminator v3.1 Cycle Sequencing Kit and the labeled DNA was purified by ethanol precipitation. The precipitate was then dissolved in Hi-Di Formamide (Applied Biosystems). DNA sequences were determined using the Applied Biosystem 3130/3130xl Genetic Analyzer (Applied Biosystems).

### Western blot analysis

Frozen tissues (approximately 0.2 g each) were homogenized using lysis buffer (10 mM Tris-HCl pH 7.4; 150 mM NaCl; 1% Triton X-100; 0.5% sodium deoxycholate; 0.1% SDS) containing a protease inhibitor cocktail (Complete EDTA-free Protease Inhibitor Cocktail, Roche Applied Science). After being mixed well, the homogenates were incubated on ice for 1 h. Thereafter, they were centrifuged at 16,000×g for 20 min at 4 °C and the supernatants were collected. The protein content in the supernatants was measured by using the DC Protein Assay Kit (Bio-Rad Laboratories, Japan) and 10 μg of protein was subjected to SDS-PAGE on a 10% acrylamide gel. Subsequently, the proteins in the gel were transferred on PVDF membranes (Hybond P; GE Healthcare, Little Chalfont, Buckinghamshire, UK) by semidry blotting. The membrane was incubated for 30 min with EzBlock Chemi (ATTO Corporation, Tokyo, Japan). The membrane was then washed with TBS buffer supplemented with 0.05% Tween 20 (TBS-T) and incubated overnight at 4 °C with the primary antibody, rabbit polyclonal anti-mouse IAH1 (1:20,000; Medical and Biological laboratories, Japan) and anti-mouse alpha-Tubulin (1:3,000; #2144; Cell Signaling Technology Inc.). Afterwards, the membrane was washed with TBS-T and then incubated with the secondary antibody, horseradish peroxidase-conjugated goat anti-rabbit IgG antibody (1:10,000; #7074; Santa Cruz Biotechnology, USA), for 1 h at 20–25°C. Finally, the membrane was washed with TBS-T and the proteins bands were detected with a West Dura Western Blot Detection kit using the ECL method (Thermo Fisher Scientific). All antibodies were diluted with Can Get Signal (TOYOBO, Tokyo, Japan).

### Measurement of serum lipids and glucose concentrations

Serum triglyceride (TG), total cholesterol (TC), HDL-cholesterol (HDL-C), phospholipid (PL), and non-esterified fatty acid (NEFA) concentrations were measured using Triglyceride-E test Kit, Cholesterol-E test Kit, HDL-Cholesterol-E test Kit, Phospholipid-C test Kit, and NEFA-C test Kit (all assay kits, respectively, which were obtained from Wako Pure Chemical Industries, Japan). Serum glucose concentrations were measured using Glucose-CII test kit (Wako Pure Chemical Industries).

### Measurement of hepatic lipids

Frozen livers (approximately 0.3 g each) were homogenized with 25 mL of chloroform-methanol (2:1) and statically extracted overnight. The organic extracts (200 μL) were dried and dissolved in isopropanol (200 μL). TG and total TC in isopropanol were measured using the Triglyceride-E test Kit and Cholesterol-E test Kit, respectively. The remaining organic extract was used for the measurement of total liver lipids as previously described by Folch *et al*. [16].

### Real-time qPCR

Total RNA from tissue was extracted using the TRI reagent (Molecular Research Center Inc.). The total RNA was treated with the TURBO DNA-free kit (Thermo Fisher Scientific) to remove DNA contamination. Then, cDNA was synthesized using the High Capacity Reverse Transcription kit (Applied Biosystems). We used a StepOne Plus Real-Time PCR System (Applied Biosystems) with the Thunderbird qPCR Mix or the Thunderbird SYBR qPCR Mix (TOYOBO, Japan) in order to measure gene expression levels. Each mRNA level was normalized to the corresponding β-actin mRNA level. TaqMan probes (TaqMan Gene Expression Assays, Mm00509467_m1; Applied Biosystems) were used to determine the mRNA level of *Iah1*. The primers used for the SYBR Green assays are shown in S3 Table.

### DNA microarray analysis in epididymal fat

Total RNA was extracted from the epididymal fat of WT_A12 and KO_A12 mice fed with a high-fat diet for 12 weeks using the TRI reagent. Then, the obtained RNA was purified using the RNeasy Mini Kit (QIAGEN). Total RNA from three mice per strain was pooled for each array. The transcripts from epididymal fat were measured using a Clariom S Mouse Array (Applied Biosystems). Raw data were normalized with the STT-RMA algorithm using the Applied Biosystems GeneChip Console Software ver.1.3.0. The microarray data have been deposited in the NCBI Gene Expression Omnibus (GEO) (GSE14750).

### Statistical analysis

All results were expressed as mean ± standard error of the mean (SEM). Student’s *t*-test was used to compare the means between WT (WT_B6 or WT_A12) and KO (KO_B6 or KO_A12) mice. Differences with a *p* < 0.05 with statistically significant.

## Results

### Iah1 KO mice on a C57BL/6N background

**Fig 1:**
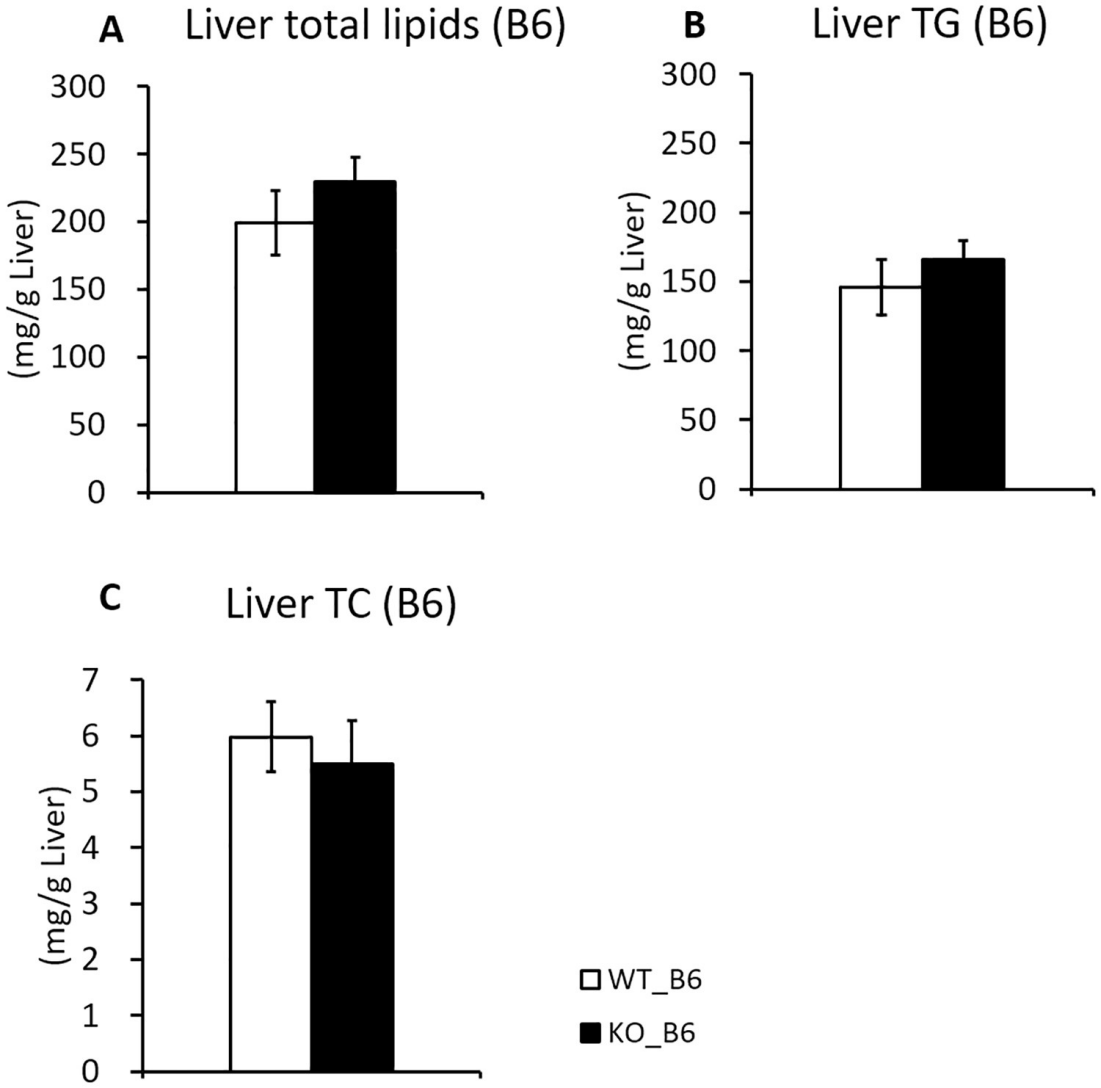
Liver TG, total lipids, and TC content of WT_B6 and KO_B6 mice. (A) Liver total lipids, (B) triglycerides, and (C) total cholesterol concentrations in WT_B6 and KO_B6 mice fed with a high-fat diet for 12 weeks. Data were expressed as mean ± SEM. WT_B6 (n = 8), KO_B6 (n = 8).

We constructed systemic *Iah1* KO mice (KO_B6) by using the Cre/loxP system (S1 Fig) and analyzed their phenotype. The expression of *Iah1* mRNA and IAH1 protein were not systemically detected in KO_B6 mice (S2 Fig). However, we did not observe a significant increase in total lipids and TG accumulation in the liver (Fig 1). Body weight, serum lipids concentrations, serum glucose concentrations, and liver weight did not differ between KO_B6 mice and WT_B6 mice (Table 1). Notably, the mesenteric fat weight was significantly increased in KO_B6 mice but the weight of other white adipose tissues remained unchanged between KO_B6 mice and WT_B6 mice (Table 1). Severe fatty liver in SMXA-5 mice is caused by a combination of multiple genes that are latent in the genomes of both A/J and SM/J mice. As such, systemic *Iah1*-KO mice on a C57BL/6N background might have not have led to the development of multiple gene-induced fatty liver. We also constructed *Iah1*-KO mice on A/J-12^SM^ background and analyzed their phenotype.

**Table 1:** Body weight, serum parameters, and tissue weight of B6-*Iah1* WT (WT_B6) and KO (KO_B6) mice fed with a high-fat diet (HFD) for 12 weeks.

	C57BL/6		
	WT_B6	KO_B6	P-value
Body weight (g)			
0 weeks of feeding with the HFD (Initial)	23.4 ± 0.7	23.2 ± 0.5	NS
12 weeks of feeding with the HFD (Final)	47.3 ± 0.6	47.8 ± 0.7	NS
Serum lipids concentration at 12 weeks of HFD			
Serum TG (mg/dL)	93 ± 5	83 ± 3	NS
Serum TC (mg/dL)	140 ± 11	141 ± 13	NS
Serum glucose (mg/dL)	210 ± 14	212 ± 22	NS
Weight of tissues (g/100 g body weight)			
Liver	4.89 ± 0.32	5.25 ± 0.35	NS
Subcutaneous fat ^a^	4.49 ± 0.20	4.77 ± 0.12	NS
Epididymal fat	3.30 ± 0.38	3.14 ± 0.26	NS
Mesenteric fat	2.94 ± 0.16	3.40 ± 0.11*	0.034
Retroperitoneal fat	1.46 ± 0.01	1.57 ± 0.05	NS

Each value is expressed as the mean ± SEM. WT_B6 (n = 8), KO_B6 (n = 8).

*p<0.05, significant difference when compared to WT_B6 by student’s *t*-test.

NS, not significant.

^a^ Subcutaneous fat was dissected between the root of the forefoot and the hind leg on the right side of the body.

### Iah1 KO mice on A/J-12^SM^ background

**Fig 2:**
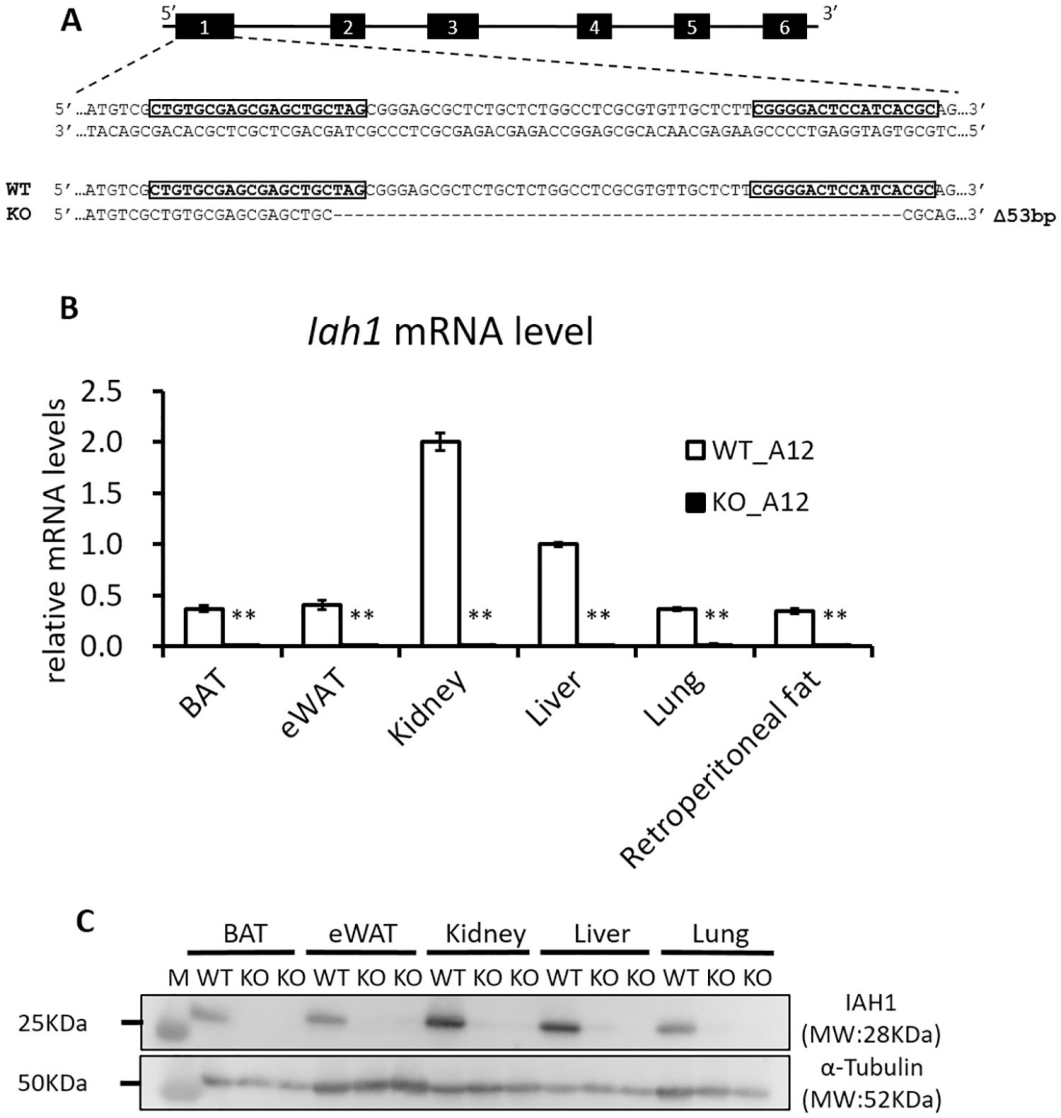
Generation of A/J-12^SM^ *Iah1* knockout (KO_A12) mice. (A) The sequence of crRNA (boxed) which targets the Cas9 nuclease to a region of exon 1 in the *Iah1* mouse gene. The mouse with a 53bp deletion and a frameshift mutation was identified. (B) Real-time qPCR analysis of *Iah1* mRNA levels in WT_A12 and KO_A12 mice. Data were expressed as mean ± SEM. WT_A12 (n = 4), KO_A12 (n = 4). **, Significantly different (*p*<0.01) when compared with WT_A12 mice by student’s *t*-test. (C) Western blot analysis of the IAH1 protein (10 μg each, 28 kDa) with α-Tubulin as a loading control in WT_A12 and KO_A12 mice. The size marker (Precision Plus Protein Standards, Bio-Rad) was loaded in lane M. Tissues were collected from WT_A12 and KO_A12 mice fed with a high-fat diet for 12 weeks. BAT, brown adipose tissue; eWAT, epididymal white adipose tissue.

We constructed *Iah1*-KO mice on an A/J-12^SM^ background (KO_A12) by using the CRISPR/Cas9 system to investigate the relationship between *Iah1* and fatty liver development. The CRISPR/Cas9 system generated a 53 bp deletion that resulted in frameshift mutations leading to the formation of a premature stop codon (Fig 2A). This mutation induces nonsense-mediated decay of the mRNA transcript. The expression of *Iah1* mRNA and IAH1 protein were not detected in A/J-12^SM^ *Iah1-*KO mice (KO_A12) (Fig 2B and 2C). Regarding the predicted off-target cleavage sites, no mutations were detected by sequencing.

**Fig 3:**
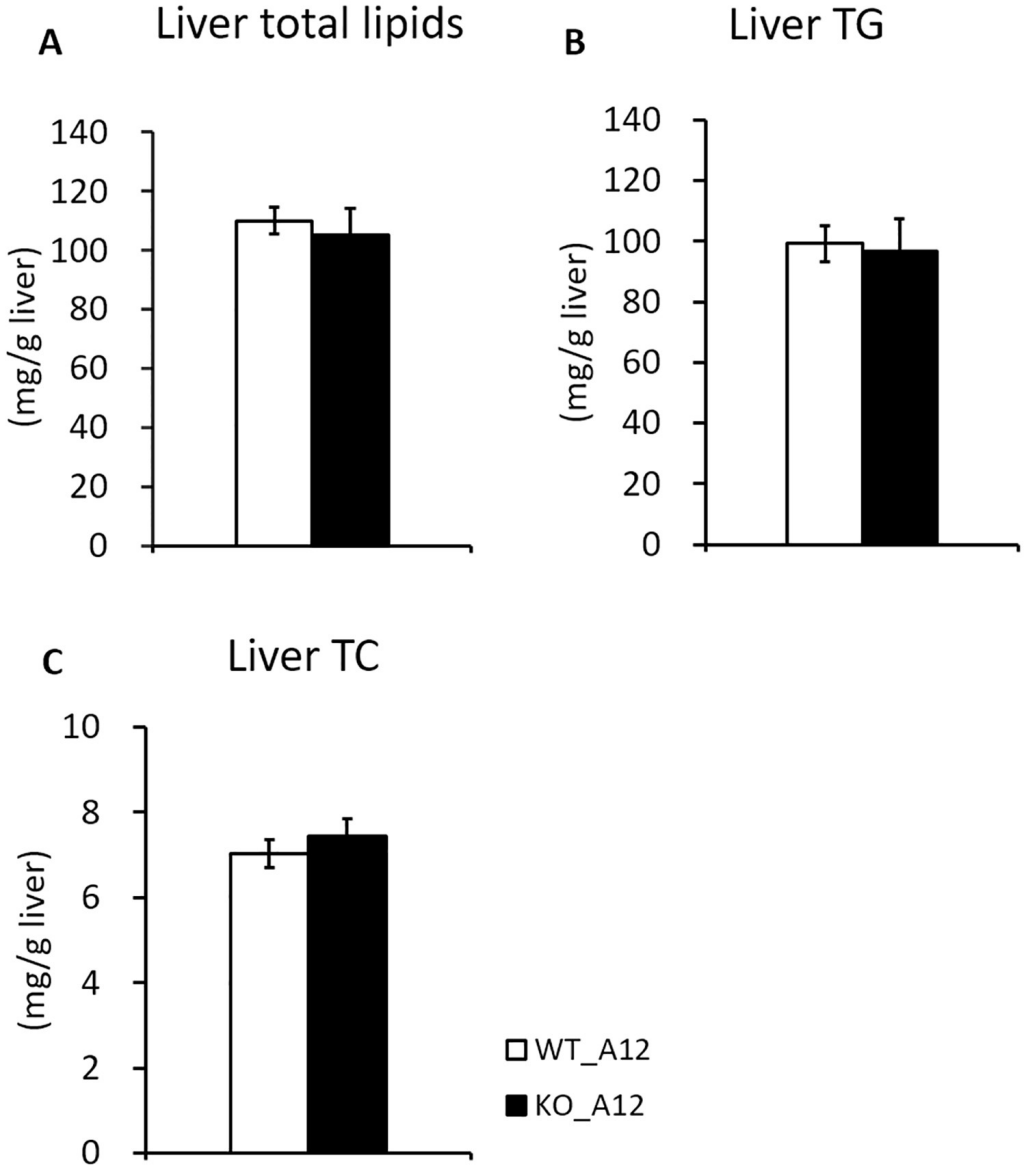
Liver TG, total lipids, and TC contents in WT_A12 and KO_A12 mice. (A) Liver total lipids, (B) triglyceride, and (C) total cholesterol concentrations in WT_A12 and KO_A12 mice fed with a high-fat diet for 12 weeks. Data were expressed as mean ± SEM. WT_A12 (n = 16), KO_A12 (n = 11).

Compared to WT_B6 mice (Table 1, Fig 1), WT_A12 mice showed lower final body weight, liver weight, and liver lipids concentration (Table 2, Fig 3). In contrast, the serum lipids concentrations and the white adipose tissue weights of WT_A12 mice were higher than those of WT_B6 mice (Tables 1 and 2). These results showed that WT_A12 mice were resistant to high-fat diet-induced fatty liver but not abdominal fat accumulation when compared to WT_B6 mice. Initial body weight (6 weeks of age) and final body weight (18 weeks of age) were not altered by *Iah1* deficiency on an A/J-12^SM^ background (Table 2). Moreover, the weight of the liver and white adipose tissue of KO_A12 mice did not differ from those found in WT_A12 mice (Table 2). Similarly, serum lipids concentrations and serum glucose concentrations at 12 weeks of feeding (18 weeks of age) did not differ between WT_A12 and KO_A12 mice (Table 2). We expected liver TG levels to increase in KO_A12 mice but our found that they remained unchanged between KO_A12 and WT_A12 mice (Fig 3B). Likewise, there were no significant changes in liver total lipid contents and liver TC between WT_A12 and KO_A12 mice (Fig 3A and 3C). Thus, these data indicate that *Iah1* deficiency does not alter body weight, serum lipids and serum glucose concentrations, and liver lipid contents.

**Table 2:** Body weight, serum parameters, and tissue weight of A/J-12^SM^ *Iah1*-WT (WT_A12) and KO (KO_A12) mice fed with a high-fat diet (HFD) for 12 weeks.

	A/J-12^SM^		
	WT_A12	KO_A12	P-value
Body weight (g)			
0 weeks of feeding with the HFD (Initial)	22.2 ± 0.8	23.7 ± 0.7	NS
12 weeks of feeding with the HFD (Final)	42.0 ± 0.7	41.0 ± 1.3	NS
Serum lipids concentrations at 12 weeks of feeding			
Serum TG (mg/dL)	120 ± 5	126 ± 6	NS
Serum TC (mg/dL)	123 ± 9	120 ± 8	NS
Serum HDL-C (mg/dL)	136 ± 5	127 ± 5	NS
Serum PL (mg/dL)	175 ± 9	167 ± 11	NS
Serum NEFA (mEq/L)	0.93 ± 0.03	0.95 ± 0.02	NS
Serum glucose (mg/dL)	189 ± 6	183 ± 12	NS
Weight of tissues (g/100 g body weight)			
Liver	3.10 ± 0.07	3.22 ± 0.08	NS
Subcutaneous fat ^a^	4.22 ± 0.08	4.33 ± 0.17	NS
Epididymal fat	6.31 ± 0.19	6.12 ± 0.28	NS
Mesenteric fat	3.24 ± 0.09	2.98 ± 0.15	NS
Retroperitoneal fat	1.61 ± 0.06	1.57 ± 0.05	NS

Each value is expressed as the mean ± SEM. WT_A12 (n = 16), KO_A12 (n = 11).

NS, not significant by student's *t*-test.

^a^ Subcutaneous fat was dissected between the root of the forefoot and the hind leg on the right side of the body.

### Iah1 deficiency does not affect lipid metabolism-related genes expression in the liver and epididymal fat

**Fig 4:**
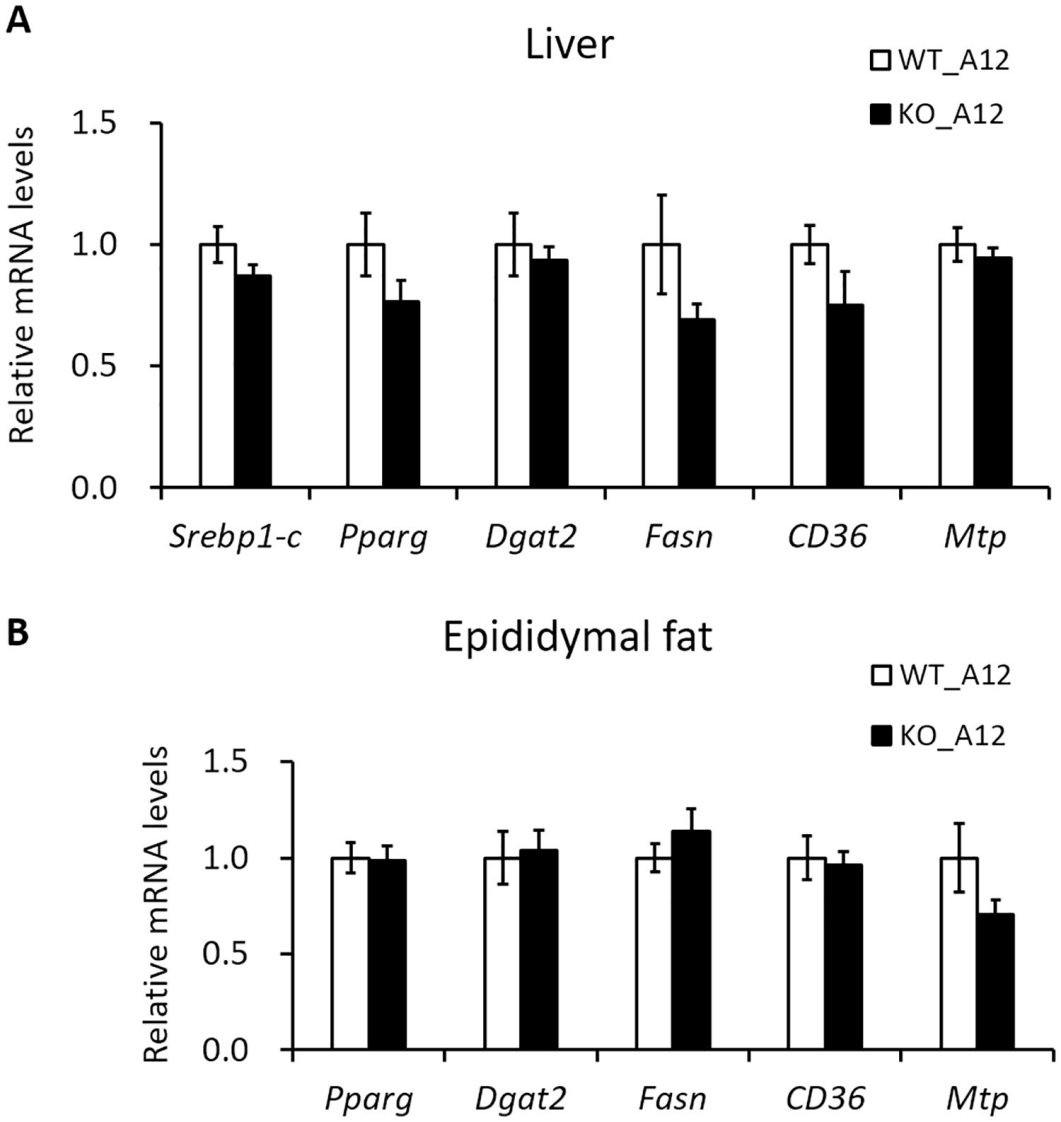
The mRNA levels of lipid metabolism-related genes in the liver and epididymal fat of WT_A12 and KO_A12 mice fed with a high-fat diet for 12 weeks. The mRNA levels were measured by real-time qPCR in the liver (A) and epididymal fat (B). Data were expressed as mean ± SEM. WT_A12 (n = 5–8), KO_A12 (n = 5–8). *Srebp1-c*, sterol regulatory element binding transcription factor 1c; *Pparγ*, peroxisome proliferator activated receptor gamma; *Dgat2*, diacylglycerol *O*-acyltransferase 2; *Fasn*, fatty acid synthase; *CD36*, Cd36 antigen; *Mtp*, microsomal triglyceride transfer protein.

We previously analyzed the mRNA levels of lipid metabolism-related genes in Hepa1-6 cells (mouse hepatoma cell line) which overexpressed mouse *Iah1*. We found that the mRNA expression of diacylglycerol *O*-acyltransferase 2 (*Dgat2*) and Cd36 antigen (*Cd36*) were suppressed in Hepa1-6 cells which overexpressed mouse *Iah1* [6]. In this study, in addition to these genes, we also measured the mRNA levels of lipid metabolism-related genes (*Srebp1-c*, *Pparγ*, *Fasn*, and *Mtp*) in the liver and epididymal fat of KO_A12 mice. Our results showed that the mRNA levels were not significantly different between WT_A12 and KO_A12 mice (Fig 4A and 4B). Therefore, this suggests that *Iah1* deficiency does not affect the expression of these lipid metabolism-related genes in the liver and epididymal fat.

### DNA microarray analysis of epididymal fat in KO_A12 mice

**Fig 5:**
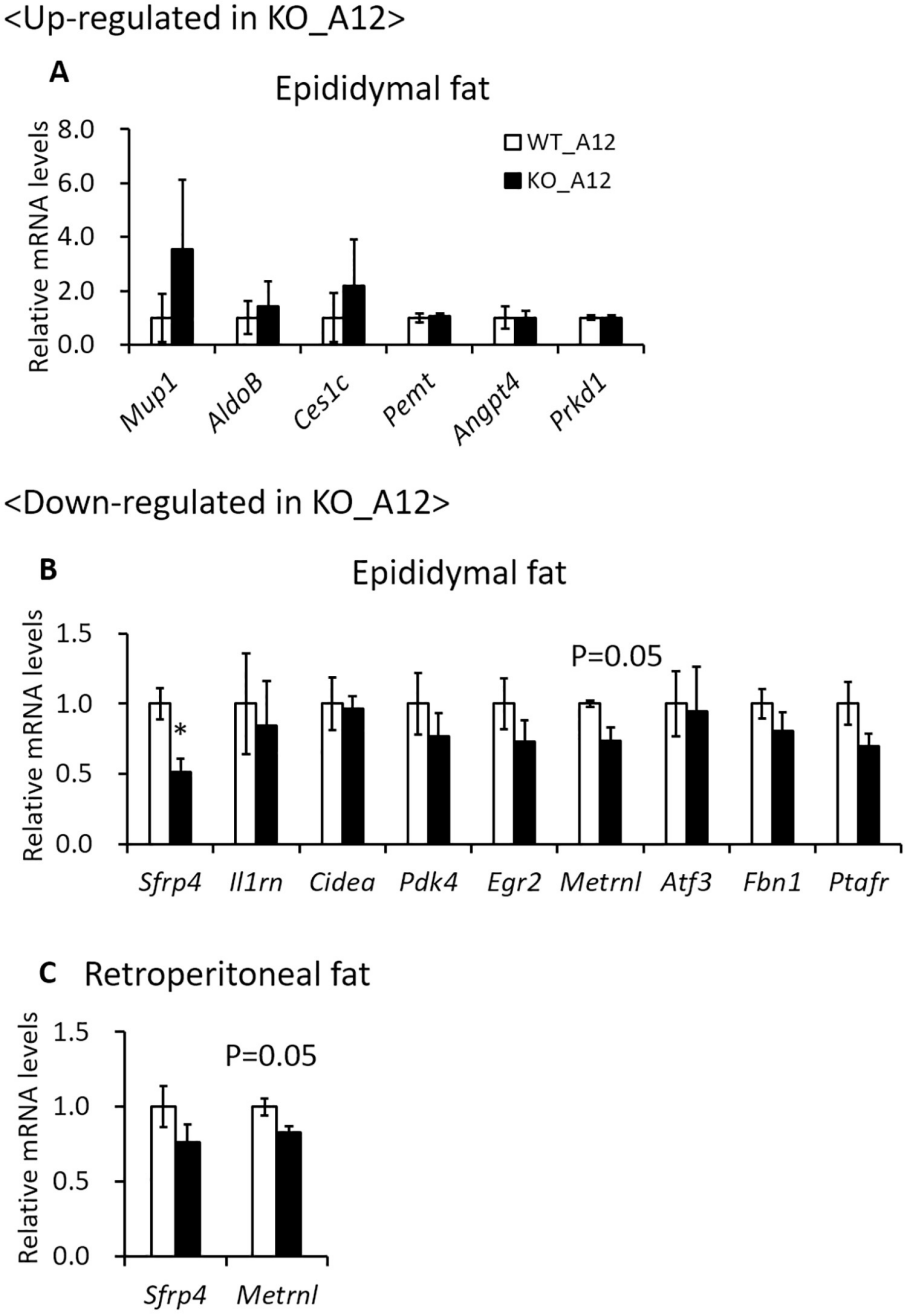
The mRNA levels of genes selected via DNA microarray analysis of epididymal fat in the epididymal and retroperitoneal fat of WT_A12 and KO_A12 mice fed with a high-fat diet for 12 weeks. The mRNA levels of upregulated or downregulated genes were measured by real-time qPCR in epididymal fat (A, B) and retroperitoneal fat (C). Data were expressed as mean ± SEM. WT_A12 (n = 5–8), KO_A12 (n = 5–8). *, Significantly different (*p* < 0.05) by student’s *t*-test when compared to the WT_A12 group. The P-value was calculated using the student’s *t*-test versus the WT_A12 group. *Mup1*, major urinary protein 1; *AldoB*, aldolase B, fructose-bisphosphate; *Ces1c*, carboxylesterase 1C; *Pemt*, phosphatidylethanolamine N-methyltransferase; *Angpt4*, angiopoietin 4; *Prkd1*, protein kinase D1; *Sfrp4*, secreted frizzled-related protein 4; *Il1rn*, interleukin 1 receptor antagonist; *Cidea*, cell death-inducing DNA fragmentation factor, alpha subunit-like effector A; *Pdk4*, pyruvate dehydrogenase kinase; *Egr2*, early growth response 2; *Metrnl*, meteorin, glial cell differentiation regulator-like; *Atf3*, activating transcription factor 3; *Fbn1*, fibrillin 1; *Ptafr*, platelet-activating factor receptor.

**Fig 6:**
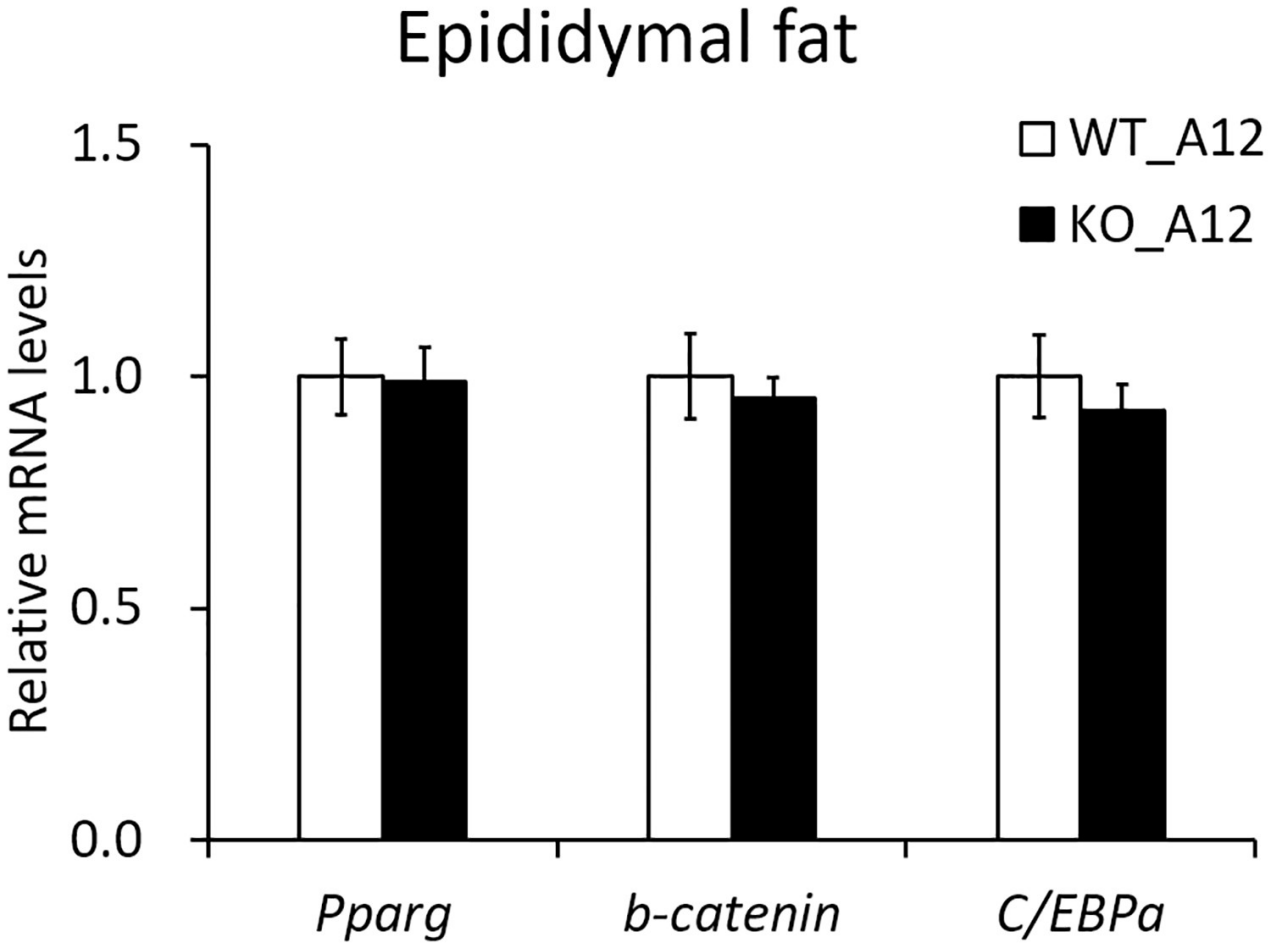
The mRNA levels of *Sfrp4*-downstream genes in the epididymal fat of WT_A12 and KO_A12 mice fed with a high-fat diet for 12 weeks. The mRNA levels of *Pparγ*, *β-catenin*, and *C/EBPα* in epididymal fat were measured by real-time qPCR. Data were expressed as mean ± SEM. WT_A12 (n = 5–8), KO_A12 (n = 5–8). *Pparγ*, peroxisome proliferator activated receptor gamma; *C/EBPα*, CCAAT/enhancer-binding protein alpha.

The kidney is the tissue where the *Iah1* gene is most abundantly expressed. The *Iah1* gene was also shown to be abundantly expressed in the liver and epididymal fat (Fig 2B and 2C). Our previous report showed that the epididymal fat weight was inversely correlated with liver weight and TG content in A/J-12^SM^-related mouse strains [7]. The adipose tissue plays an important role in storing the excessive energy as triglyceride. However, adipose tissue insulin resistance causes elevation of serum free fatty acids level by attenuating the suppression of lipolysis, leading to lipid accumulation in ectopic tissues (liver, skeletal muscle, and pancreas) [17]. Macrophage activation in adipose tissue insulin resistance is closely linked to obesity-induced NAFLD [18, 19]. In addition, adipose tissue inflammation causes reduction of lipid storage-capacity in adipose tissue and dysregulation of adipokines production [20]. Therefore, to investigate the effect of *Iah1* deficiency on adipose tissue, we performed DNA microarray analysis of epididymal fat in WT_A12 and KO_A12 mice. We identified 126 upregulated genes (> 2.0 fold and signal strength of > 128) in KO_A12 mice when compared with WT_A12 mice (S4 Table). Moreover, 167 downregulated genes (< 0.5 fold and signal strength of > 128) were identified in KO_A12 mice when compared with WT_A12 mice (S5 Table). Of these genes, we selected sixty genes which were known to be associated with glucose or lipid metabolism and were expressed in white adipose tissue. The mRNA levels of upregulated genes (*Mup1*, *AldoB*, *Ces1c*, *Pemt*, *Angpt4*, and *Prkd1*) and downregulated genes (*Sfrp4*, *Il1rn*, *Cidea*, *Pdk4*, *Egr2*, *Metrnl*, *Atf3*, *Fbn1*, and *Ptafr*) in the epididymal fat of KO_A12 mice were measured via real-time qPCR (S4 and S5 Tables). Secreted frizzled-related protein 4 (*Sfrp4*) mRNA levels in epididymal fat were found to be significantly decreased in KO_A12 mice (Fig 5B). Moreover, Meteorin like, glial cell differentiation regulator (*Metrnl*) mRNA levels tended to be slightly decreased in KO_A12 mice when compared to WT_A12 mice (*p* = 0.05) (Fig 5B). The changes in gene expression for the other analyzed genes were not confirmed by real-time qPCR. Therefore, we also measured *Sfrp4* and *Metrnl* mRNA levels in the retroperitoneal adipose tissue, which is a type of visceral white adipose tissue. Although *Metrnl* mRNA levels tended to be slightly decreased in KO_A12 mice (*p* = 0.05), there were no significant changes in *Sfrp4* mRNA levels in the retroperitoneal adipose tissue (Fig 5C). These results indicated that *Iah1* deficiency in visceral white adipose tissue (including epididymal and retroperitoneal fat) decreased *Sfrp4* and *Metrnl* gene expression. Decreased *Sfrp4* mRNA levels may to a decrease in the mRNA levels of downstream genes (*Pparγ* and *C/EBPα*) and increase *β-catenin* mRNA levels. However, significant differences in the expression of these genes were not observed (Fig 6).

## Discussion

In this study, we focused on *Iah1* gene as the most likely candidate gene for *Fl1sa*, a QTL for fatty liver on chromosome 12 identified in the high-fat diet-induced fatty liver model mouse, SMXA-5 [6]. Therefore, in order to investigate the relationship between *Iah1* and fatty liver *in vivo*, we analyzed the phenotype of *Iah1*-KO mice established on two different backgrounds (C57BL/6N and A/J-12^SM^).

KO_B6 mice were systemically deficient in *Iah1* mRNA and IAH1 protein, and *Iah1* gene deficiency did not lead to embryonic death. Moreover, we did not observe a significantly higher TG accumulation in KO_B6 mice when compared with WT_B6 mice, both of which were fed with a high-fat diet for 12 weeks. A previous study has reported that C57BL/6J mice, which is one of the C57BL/6-derived substrains, are more likely to develop severe obesity and type 2 diabetes than A/J mice fed with a high-fat diet [21]. Therefore, we thought that the effect of *Iah1* deficiency on fatty liver development might be masked in the C57BL/6N background. As such, we created *Iah1*-KO mice on an A/J-12^SM^ background using the CRISPR/Cas9 system. We previously reported that liver TG accumulation in A/J-12^SM^ mice which had the SM/J allele on *Fl1sa* was suppressed when compared with A/J mice [5]. We expected that *Iah1* deficiency on an A/J-12^SM^ background would significantly induce fatty liver development. However, liver TG accumulation in KO_A12 mice did not differ from that in WT_A12 (A/J-12^SM^) mice (Fig 3). Altogether, the abovementioned data demonstrated that *Iah1* is not a gene responsible for *Fl1sa*.

Reduction of lipid storage-capacity and dysregulation of secretion adipokines in adipose tissue are involved in the development of fatty liver [17, 20]. Therefore, we performed DNA microarray analysis of epididymal fat in *Iah1* deficiency on an A/J-12^SM^ background, and the data showed that *Iah1* deficiency *in vivo* might decrease adipokines (*Sfrp4* and *Metrnl*) gene expression in epididymal fat (Fig 5). *Sfrp4* is an adipokine and a member of the *Sfrp* family of proteins, which regulate the activity of the Wingless-type (Wnt) signaling pathway. *Sfrp4* inhibits Wnt from binding to the Frizzled receptor and leads to β-catenin degradation. Wnt signaling maintains preadipocytes in an undifferentiated state by suppressing the expression of adipogenic transcription factors (*C/EBPα* and *Pparγ*) [22]. Horbelt *et al*. reported that *Sfrp4* mRNA levels were high in the visceral adipose tissue of obese or type 2 diabetes patients and that *Sfrp4* promotes *de novo* lipogenesis in mice hepatocytes [23]. These results suggest that a decrease in adipokine *Sfrp4* levels in the epididymal fat of KO_A12 mice activates the Wnt signaling pathway and leads to inhibition of lipid synthesis in the liver. However, in the epididymal fat of KO_A12 mice, the expression of *Sfrp4* downstream genes (*β-catenin*, *C/EBPα*, and *Pparγ*) remained unchanged (Fig 6). *Metrnl* (also known as *Subfatin* and *Cometin*) promotes differentiation of adipocytes by increasing the expression of lipid metabolism genes [24]. *Metrnl* adipocyte-specific KO mice under a high-fat diet showed insulin resistance [24]. Moreover, an increase in circulating levels of *Metrnl* in mice by intravenous injections of adenoviral vectors stimulates thermogenesis in beige fat and improves glucose tolerance [25]. However, the present study did not confirm the changes of *Sfrp4* and *Metrnl* protein levels in adipose tissue and serum.

As *Iah1* is not a gene responsible for *Fl1sa*, we take into consideration another candidate gene, namely, *Lpin1* (encoding the Lipin1 protein) which is located at 16.7 Mb on mouse chromosome 12. *Lipin1* was identified as a mutated gene in the fatty liver dystrophy (*fld*) mouse [26]. The mutated *lipin1* gene was shown to impair adipose tissue development, which led to fatty liver development. Phan and Reue reported that modulation of *lipin1* expression levels leads to dramatic alterations in adiposity [27]. Therefore, it is necessary to analyze other candidate genes for *Fl1sa*, including *Lipin1*, to elucidate the mechanism of fatty liver development in SMXA-5 mice.

## Conclusions

In this study, to clarify the relationship between the *Iah1* gene (most likely candidate gene for *Fl1sa*) and fatty liver *in vivo*, we constructed *Iah1*-KO mice on two different genetic backgrounds (C57BL/6N and A/J-12^SM^). Data from both types of *Iah1*-KO mice (KO_B6 and KO_A12) demonstrated that the absence of the *Iah1* gene did not affect lipid accumulation in the liver. We conclude that the *Iah1* gene is not a gene responsible for *Fl1sa*.

## Supporting information

S1 Fig Generation of C57BL/6 *Iah1* knockout (KO_B6) mice. (A) The scheme of KO_B6 mice generation by using the Cre-loxP system. Figure modified from Skarnes *et al*. [11] and EUCOMM (http://www.mousephenotype.org/about-ikmc/eucomm). The tm1a mice (C57BL/6NTac genetic background) have a heterozygous knockout-first allele with the loxP sites and Frt sites. The CAG-Cre transgenic mice (C57BL/6NCrSlc genetic background) show constitutive expression of the Cre recombinase gene under the control of the CAG promoter. The tm1b mice have the genome construct of *Iah1* which has exon 3 and 4 deleted. (B) Breeding scheme of KO_B6 mice.
(PDF)

S2 Fig Expression levels of *Iah1* mRNA and IAH1 protein in KO_B6 mice. (A) Real-time qPCR analysis of *Iah1* mRNA levels in WT_B6 and KO_B6 mice. Data were expressed as mean ± SEM. (n = 4–7, **p<0.01 versus B6_WT mice by student’s *t*-test). The mRNA levels were measured by real-time qPCR. (B) Western blot analysis of the IAH1 protein (28 kDa) with α-tubulin as a loading control in WT_B6 and KO_B6 mice. The size marker (Precision Plus Protein Standards, Bio-Rad) was loaded into lane M. Tissues were collected from WT_B6 and KO_B6 mice fed with a high-fat diet for 12 weeks. BAT, brown adipose tissue; eWAT, epididymal white adipose tissue.
(PDF)

S1 Table Sequences of primers used for genotyping of WT_B6 and KO_B6 mice. (DOCX)

S2 Table The off-target candidate sites ranked by MIT off-target score (>0.45) and the sequences of primers used for sequence analysis. (DOCX)

S3 Table Sequences of primers used for real-time qPCR. (DOCX)

S4 Table Upregulated genes in the epididymal fat of A/J-12^SM^ *Iah1*-KO (KO_A12) mice. (DOCX)

S5 Table Downregulated genes in the epididymal fat of A/J-12^SM^ *Iah1*-KO (KO_A12) mice. (DOCX)

S1 Raw images (PDF)

S2 Raw images (PDF)
