# Supplementary figures and images for: Ablation of Iah1, a candidate gene for diet-induced fatty liver, does not affect liver lipid accumulation in mice
Source: PLoS One. 2020 May 14;15(5):e0233087. doi: 10.1371/journal.pone.0233087 (PMC7224509; doi:10.1371/journal.pone.0233087)

A

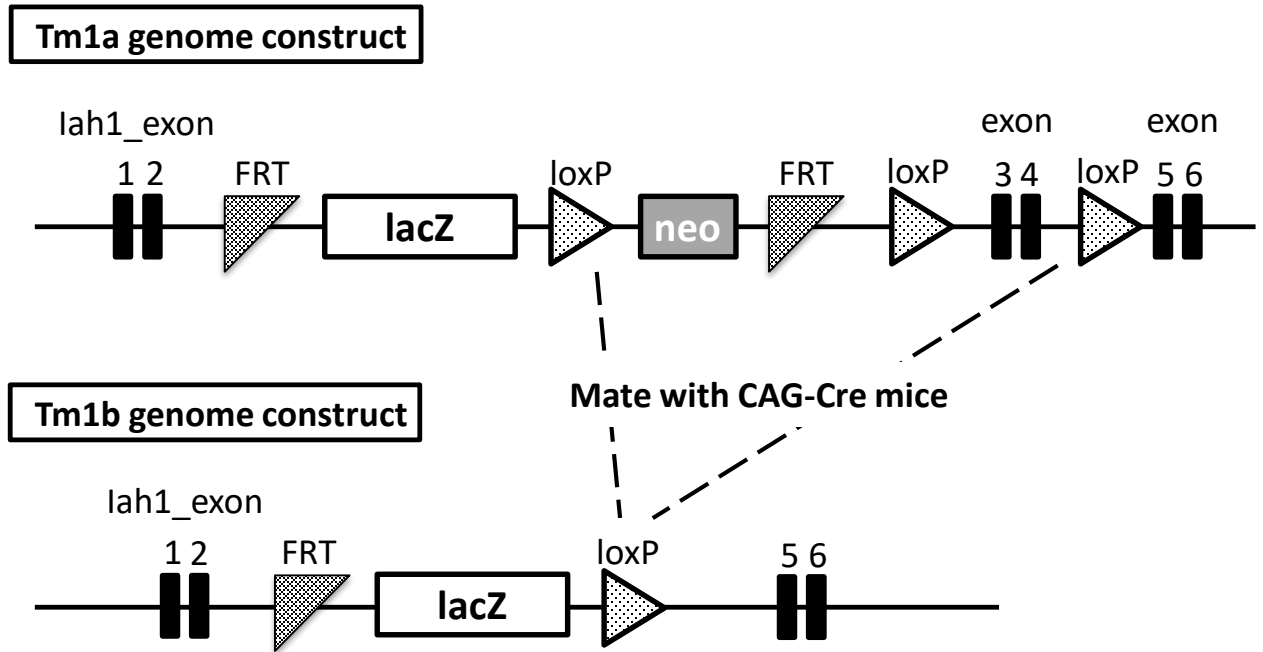

B

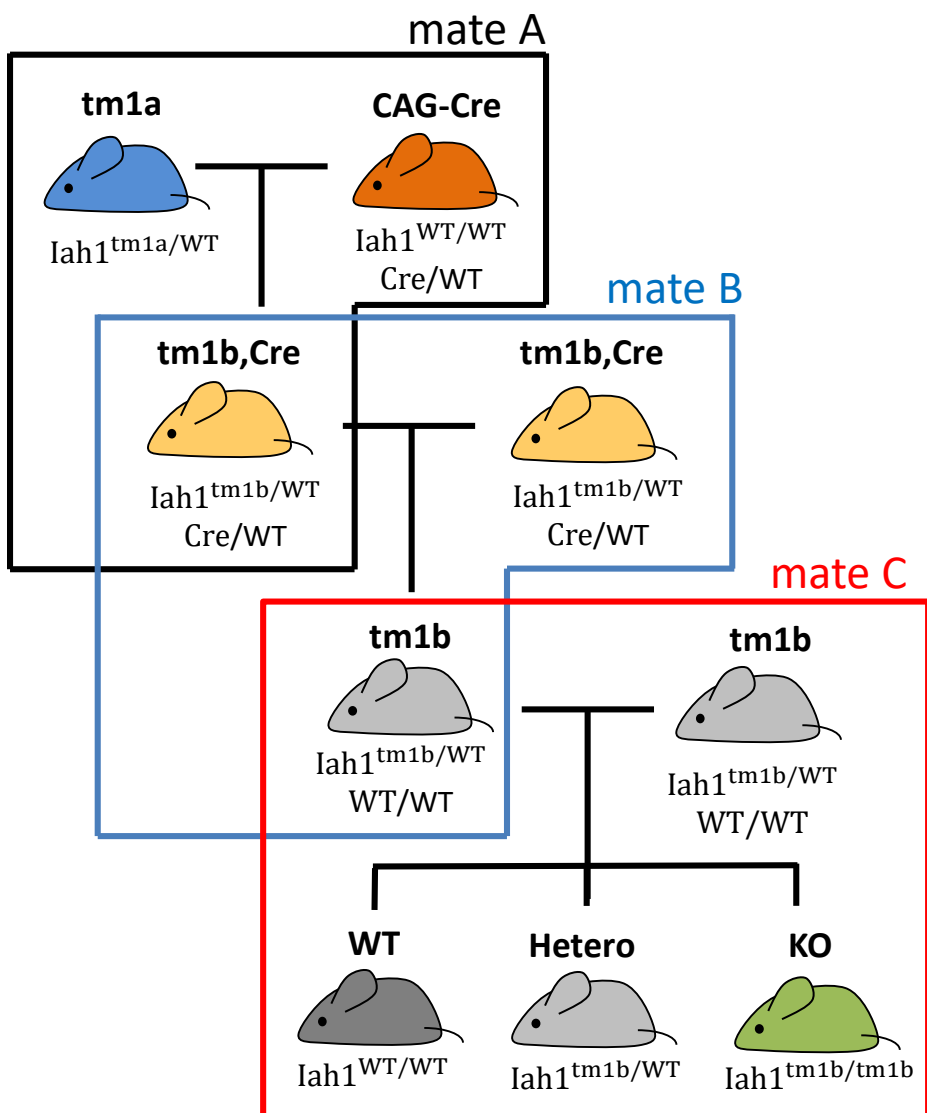

Supplement: S1 Fig — (A) The scheme of KO_B6 mice generation by using the Cre-loxP system. Figure modified from Skarnes et al. [11] and EUCOMM (http://www.mousephenotype.org/about-ikmc/eucomm). The tm1a mice (C57BL/6NTac genetic background) have a heterozygous knockout-first allele with the loxP sites and Frt sites. The CAG-Cre transgenic mice (C57BL/6NCrSlc genetic background) show constitutive expression of the Cre recombinase gene under the control of the CAG promoter. The tm1b mice have the genome construct of Iah1 which has exon 3 and 4 deleted. (B) Breeding scheme of KO_B6 mice. (PDF) [file pone.0233087.s001.pdf]

# A

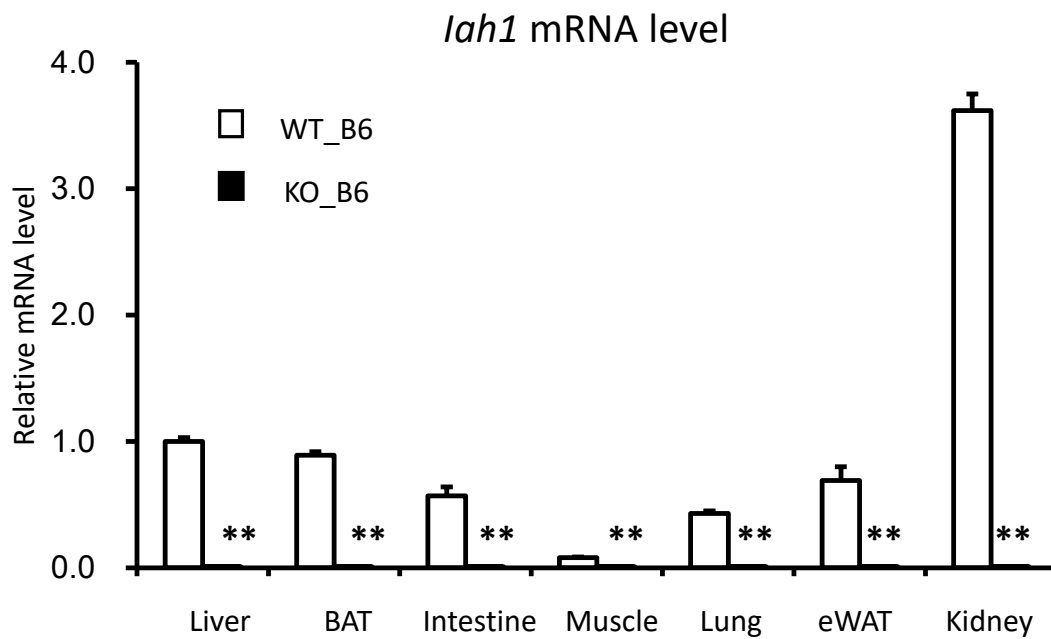

## B

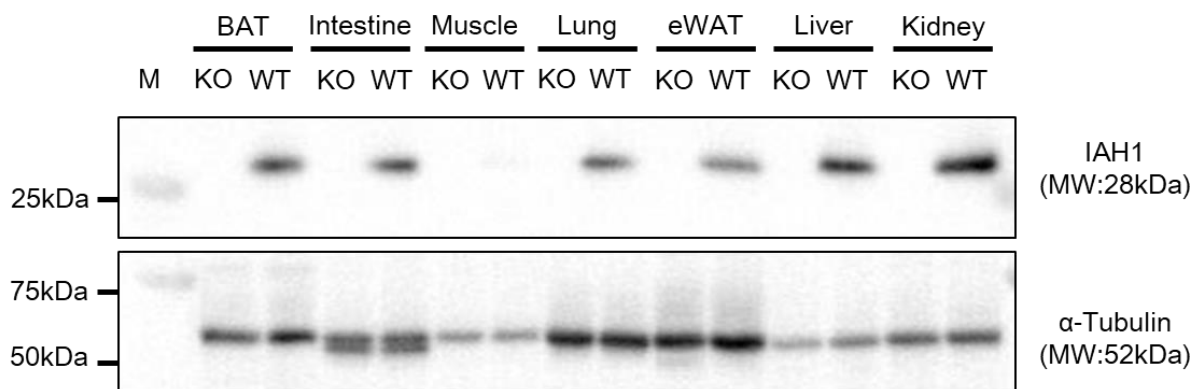

Supplement: S2 Fig — (A) Real-time qPCR analysis of Iah1 mRNA levels in WT_B6 and KO_B6 mice. Data were expressed as mean ± SEM. (n = 4–7, **p<0.01 versus B6_WT mice by student’s t-test). The mRNA levels were measured by real-time qPCR. (B) Western blot analysis of the IAH1 protein (28 kDa) with α-tubulin as a loading control in WT_B6 and KO_B6 mice. The size marker (Precision Plus Protein Standards, Bio-Rad) was loaded into lane M. Tissues were collected from WT_B6 and KO_B6 mice fed with a high-fat diet for 12 weeks. BAT, brown adipose tissue; eWAT, epididymal white adipose tissue. (PDF) [file pone.0233087.s002.pdf]

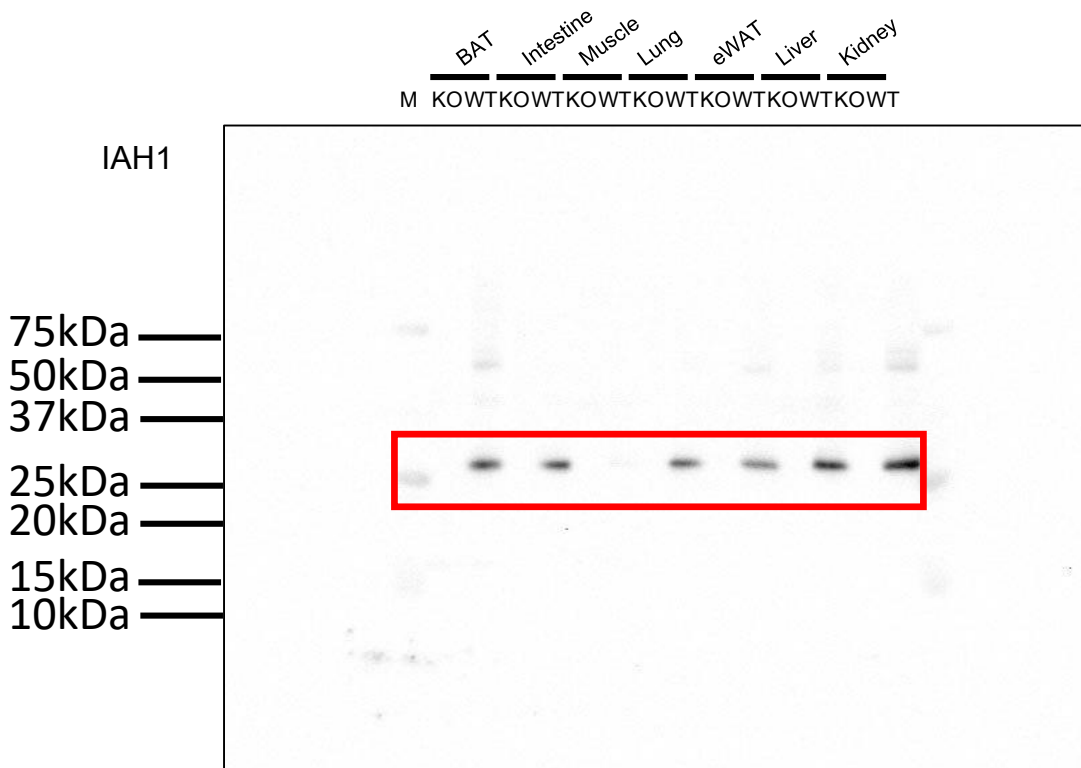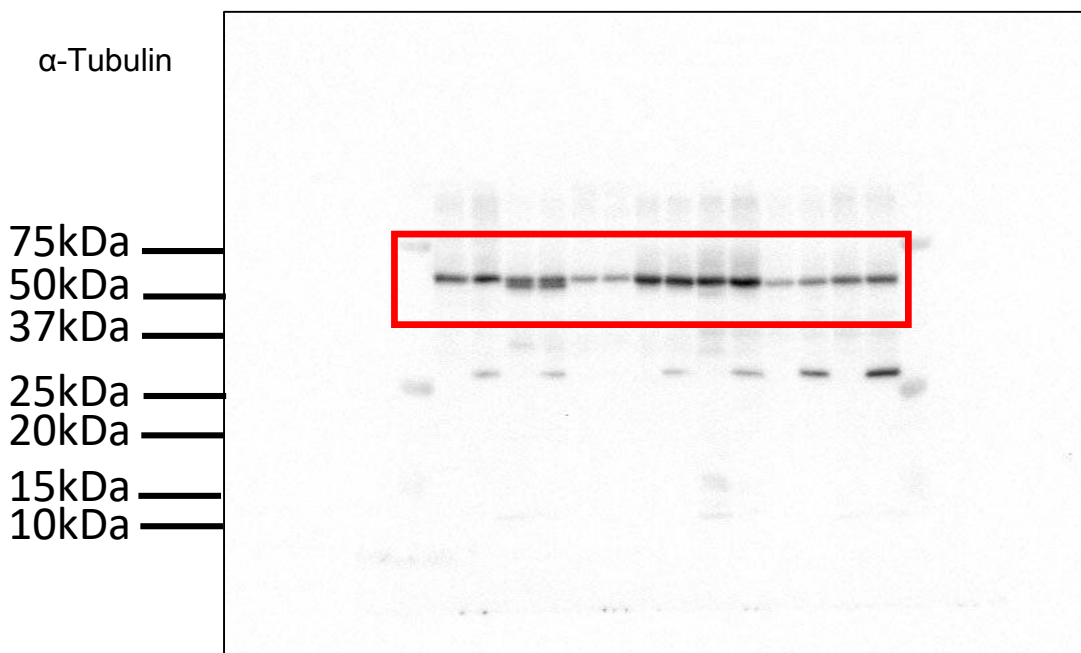

Supplement: S2 Raw images — (PDF) [file pone.0233087.s009.pdf]
