# Supplementary material for: Ablation of Iah1, a candidate gene for diet-induced fatty liver, does not affect liver lipid accumulation in mice
Source: PLoS One. 2020 May 14;15(5):e0233087. doi: 10.1371/journal.pone.0233087 (PMC7224509; doi:10.1371/journal.pone.0233087)
Supplement: S1 Table — (DOCX) [file pone.0233087.s003.docx]

**S1 Table**

**Sequences of primers used for genotyping of WT_B6 and KO_B6 mice.**

| Sequence name | Primer sequence | |
| --- | --- | --- |
| Iah1-WT | Forward:  Reverse: | CGTTGCTGGCTGACAGACTA  AGATTGTAACGGCCACTGGG |
| tm1a | Forward:  Reverse: | ATCCGGGGGTACCGCGTCGAG  ACTGATGGCGAGCTCAGACC |
| tm1b | Forward:  Reverse: | CGGTCGCTACCATTACCAGT  ACTGATGGCGAGCTCAGACC |
| LacZ | Forward:  Reverse: | ATCACGACGCGCTGTATC  ACATCGGGCAAATAATATCG |
| CAG-Cre | Forward:  Reverse: | CCTACAGCTCCTGGGCAACGTGC  CTAATCGCCATCTTCCAGCAGG |
| FLP | Forward:  Reverse: | CCTACAGCTCCTGGGCAACGTGC  CTGCTTCTTCCGATGATTCG |
