# Supplementary material for: Ablation of Iah1, a candidate gene for diet-induced fatty liver, does not affect liver lipid accumulation in mice
Source: PLoS One. 2020 May 14;15(5):e0233087. doi: 10.1371/journal.pone.0233087 (PMC7224509; doi:10.1371/journal.pone.0233087)
Supplement: S2 Table — (DOCX) [file pone.0233087.s004.docx]

**S2 Table**

**The off-target candidate sites ranked by MIT off-target score (>0.45) and the sequences of primers used for sequence analysis.**

crRNA1

| off-target sequence | MIT Off-target Score | location | primer | |
| --- | --- | --- | --- | --- |
| CTGTCTGAGCCAGCTGCTAGGGG | 0.854 | intron:Kcnt2 | Forward: | ACCAGCCCTAGCACATCAAG |
|  |  |  | Reverse: | CAAGAGATGCAGCGATTGAA |
| GTGGGGGAGGGAGCTGCTAGTGG | 0.797 | intron:Ctdspl | Forward: | TTGAAAGCAGGCGGAGTAGA |
|  |  |  | Reverse: | GCCGTGTTCTGCTTTGTGTA |
| TTGTGAGAAGGAGCTGCTAGTGG | 0.487 | intergenic:Cacng7-Cacng8 | Forward: | AGCCTCCAAACAGTGACTCA |
|  |  |  | Reverse: | ATTCCTTCAGACACTGCCCA |
| CTGGCTGAGCAAGCTGCTAGGGG | 0.458 | intergenic:Tbc1d22a-Gm23416 | Forward: | TGATTCTGTGAGGTGCCATC |
|  |  |  | Reverse: | TCCCTTGTTTGTGCTGTCTT |
| CTGGTGGAGCTAGCTGCTAGAGG | 0.458 | intergenic:Gm24462-C230034O21Rik | Forward: | GGCTTTATCATGGAGTGGTCA |
|  |  |  | Reverse: | AGAGACCAGTTTCAAGCACCA |

crRNA2

| off-target sequence | MIT Off-target Score | location | primer | |
| --- | --- | --- | --- | --- |
| TGTCTGGGACCCCATCACGCTGG | 0.794 | exon:Pxn | Forward: | CCAAAGGATGAGATGGGAGC |
|  |  |  | Reverse: | CTCCAGATACCCCGACCTC |
| CTTGGGCCGCTCCATCACGCAGG | 0.545 | intergenic:Fam155a-Gm10217 | Forward: | GGAGACCAGGCATTTTATGG |
|  |  |  | Reverse: | ATCTCTTCCGAGGCTCAAGG |
| TGTCCGGGACTCCATCACGCCGA | 0.485 | intron:2010107E04Rik | Forward: | CCTGGACAACTTCTGTGAGGA |
|  |  |  | Reverse: | CTGGGCTTTTTGCTGTGTTC |
| CTTGTGGGAATCCATCACTCAGG | 0.459 | intergenic:Pard3-mmu-mir-21c | Forward: | TTCACCCTCCAGAAAGACCC |
|  |  |  | Reverse: | TCTAAAGCAGCCACACCAGA |
