# Supplementary material for: Ablation of Iah1, a candidate gene for diet-induced fatty liver, does not affect liver lipid accumulation in mice
Source: PLoS One. 2020 May 14;15(5):e0233087. doi: 10.1371/journal.pone.0233087 (PMC7224509; doi:10.1371/journal.pone.0233087)
Supplement: S3 Table — (DOCX) [file pone.0233087.s005.docx]

**S3 Table**

**Sequences of primers used for real-time qPCR.**

| Symbol |  | Primer sequence | Accession ID |
| --- | --- | --- | --- |
| *Aldob* | Forward: | TGGTGGGCATCAAGTTGGA | NM_144903 |
|  | Reverse: | GGTGGTTTCCTTGTTTGTTCCT |  |
| *Angpt4* | Forward: | CAGCATCCGGTTTCCTTAAAGA | NM_009641 |
|  | Reverse: | AGCGCTTGATCTCTGCACAGT |  |
| *Atf3* | Forward: | GAGCAGGCAGGAGCATCCT | NM_007498 |
|  | Reverse: | GCCGGGATGATAAGGCCTAA |  |
| *β-actin* | Forward: | AGATGACCCAGATCATGTTTGAGA | NM_007393 |
|  | Reverse: | CACAGCCTGGATGGCTACGT |  |
| *β-catenin* | Forward: | CCTAGCTGGTGGACTGCAGAA | NM_001165902 |
|  | Reverse: | CACCACTGGCCAGAATGATGA |  |
| *Cd36* | Forward: | AATAGTAGAACCGGGCCAC | NM_001159558 |
|  | Reverse: | CCAACTCCCAGGTACAATCA |  |
| *C/EBPα* | Forward: | TTGAAGCACAATCGATCCATCC | NM_001287523 |
|  | Reverse: | GCACACTGCCATTGCACAAG |  |
| *Ces1c* | Forward: | CAGTCCTGCCGATTTGACAA | NM_007954 |
|  | Reverse: | CCTCCATGGATCCACACCAT |  |
| *Cidea* | Forward: | ATCACAACTGGCCTGGTTACG | NM_007702.2 |
|  | Reverse: | TACTACCCGGTGTCCATTTCT |  |
| *Dgat2* | Forward: | CCTTCCTGGTGCTAGGAGTG | NM_026384.3 |
|  | Reverse: | CCAGTCAAATGCCAGCCA |  |
| *Egr2* | Forward: | CTACCACCCTTCCCTGTTCCT | NM_010118 |
|  | Reverse: | GGCTCCATCAAGGTCCTTTG |  |
| *Fasn* | Forward: | GGGTTCTAGCCAGCAGAGTC | NM_007988 |
|  | Reverse: | TCAGCCACTTGAGTGTCCTC |  |
| *Fbn1* | Forward: | ACAATTGTTCACCGAGTCGATCT | NM_007993 |
|  | Reverse: | ACTGTACCTGGGTGTTGCCATT |  |
| *Iah1* | Forward: | AAGGGCCTGGGATGGAAA | NM_026347.3 |
|  | Reverse: | GAGTCATTGGCACCAAAGAAGAT |  |
| *Il1rn* | Forward: | CTGCACTCAATGCCACACAAT | NM_001039701 |
|  | Reverse: | GTTGGGTGGAGCCGTCTGT |  |
| *Metrnl* | Forward: | CCACTGTTGCTTTCTGCATCTG | NM_144797 |
|  | Reverse: | GGGAAACTCAGGGTCAAAGGA |  |
| Symbol |  | Primer sequence | Accession ID |
| *Mtp1* | Forward: | GCTCCCTCAGCTGGTGGAT | NM_001163457.1 |
|  | Reverse: | CAGGATGGCTTCTAGCGAGTCT | NM_008642.2 |
| *Mup1* | Forward: | CAGACAGACAATCCTATTCCCTACC | NM_001163010 |
|  | Reverse: | GTCAGAGGCCAGGATAATAGTATGCCATTC |  |
| *Pdk4* | Forward: | CGGTGCAGCTGGTGAAGAG | NM_013743 |
|  | Reverse: | CATGGAACTCCACCAAATCCA |  |
| *Pemt* | Forward: | TCTGCATCCTGCTTTTGAACA | NM_008819 |
|  | Reverse: | GGCTCATCATAGCCTGTGTGAA |  |
| *Pparγ* | Forward: | GCCCACCAACTTCGGAATC | NM_011146.3 |
|  | Reverse: | TGCGAGTGGTCTTCCATCAC |  |
| *Prkd1* | Forward: | GCCCTTCAACGAGCAACAAC | NM_008858 |
|  | Reverse: | CGCTTCGTGTGCTTCACAGA |  |
| *Ptafr* | Forward: | GATACACGCTCTTTCCGATTGTT | NM_001081211 |
|  | Reverse: | ACATAGCCGTTGGCAACCA |  |
| *Sfrp4* | Forward: | AAGCCGACCCTGGCAACATA | NM_016687 |
|  | Reverse: | TTGTGACCTCATTGCAACCACTC |  |
| *Srebp1-c* | Forward: | ACGGAGCCATGGATGCAC | NM_011480.3 |
|  | Reverse: | TGTCTCACCCCCAGCATAG |  |
