# Supplementary material for: Ablation of Iah1, a candidate gene for diet-induced fatty liver, does not affect liver lipid accumulation in mice
Source: PLoS One. 2020 May 14;15(5):e0233087. doi: 10.1371/journal.pone.0233087 (PMC7224509; doi:10.1371/journal.pone.0233087)
Supplement: S4 Table — (DOCX) [file pone.0233087.s006.docx]

**S4 Table. Upregulated genes in the epididymal fat of A/J-12^SM^ *Iah1*-KO (KO_A12) mice.**

| Gene Symbol | | Gene name | WT: Signal | KO: Signal | Fold change^a^ | Chr^b^ | Start | Gene Accession |
| --- | --- | --- | --- | --- | --- | --- | --- | --- |
| Mup12 | * | major urinary protein 12 (Mup12). | 6.99 | 15.06 | 8.07 | chr4 | 60737381 | NM_001199995 |
| Svs5 |  | seminal vesicle secretory protein 5 (Svs5). | 4.25 | 12.25 | 8.00 | chr2 | 164332740 | NM_009301 |
| Svs2 |  | seminal vesicle secretory protein 2 (Svs2). | 5.01 | 12.96 | 7.96 | chr2 | 164235929 | NM_017390 |
| Alb |  | albumin (Alb). | 8.22 | 15.99 | 7.76 | chr5 | 90460889 | NM_009654 |
| Mup1 | * | major urinary protein 1 (Mup1), transcript variant 3. | 7.03 | 14.78 | 7.75 | chr4 | 60498012 | NM_001163010 |
| Gm2083 |  | major urinary protein LOC100048885 (Gm2083). | 7.08 | 14.58 | 7.50 | chr4 | 60807821 | NM_001134644 |
| Mup2 | * | major urinary protein 2 [gene_biotype:protein_coding transcript_biotype:protein_coding] | 7.02 | 14.51 | 7.49 | chr4 | 60135913 | ENSMUST00000074700 |
| Mup19 | * | major urinary protein 19 (Mup19). | 7.48 | 14.65 | 7.17 | chr4 | 61778324 | NM_001135127 |
| Serpina1c |  | serine (or cysteine) peptidase inhibitor, clade A, member 1C (Serpina1c). | 5.22 | 12.39 | 7.17 | chr12 | 103894926 | NM_009245 |
| Mup8 | * | major urinary protein 8 (Mup8). | 7.29 | 14.37 | 7.08 | chr4 | 60218621 | NM_001134676 |
| Mup2 | * | major urinary protein 2 (Mup2), transcript variant 2. | 7.54 | 14.42 | 6.88 | chr4 | 60578241 | NM_001045550 |
| Mup7 | * | major urinary protein 7 (Mup7). | 6.95 | 13.81 | 6.86 | chr4 | 60066469 | NM_001134675 |
| Sva |  | seminal vesicle antigen (Sva). | 3.60 | 10.24 | 6.65 | chr6 | 42038394 | NM_009299 |
| Mup13 |  | major urinary protein 13 (Mup13). | 7.87 | 14.48 | 6.61 | chr4 | 61224307 | NM_001134674 |
| Svs4 |  | seminal vesicle secretory protein 4 (Svs4). | 3.73 | 9.78 | 6.05 | chr2 | 164275952 | NM_009300 |
| Ttr | * | transthyretin (Ttr). | 4.69 | 10.47 | 5.78 | chr18 | 20665250 | NM_013697 |
| Svs6 |  | seminal vesicle secretory protein 6 (Svs6). | 3.32 | 8.68 | 5.36 | chr2 | 164316747 | NM_013679 |
| A630095E13Rik |  | RIKEN cDNA A630095E13 gene (A630095E13Rik). | 4.50 | 9.75 | 5.25 | chr9 | 36635754 | NM_001033325 |
| Mup3 | * | major urinary protein 3 (Mup3). | 5.39 | 10.55 | 5.16 | chr4 | 62083476 | NM_001039544 |
| Mup21 |  | major urinary protein 21 (Mup21). | 4.66 | 9.81 | 5.15 | chr4 | 62147832 | NM_001009550 |
| Spinkl |  | serine protease inhibitor, Kazal type-like (Spinkl). | 4.56 | 9.46 | 4.89 | chr18 | 44166358 | NM_183123 |
| Serpina1a |  | serine (or cysteine) peptidase inhibitor, clade A, member 1A (Serpina1a), transcript variant 2. | 7.09 | 11.95 | 4.85 | chr12 | 103853295 | NM_001252569 |
| Fgg |  | fibrinogen gamma chain (Fgg). | 3.38 | 8.20 | 4.82 | chr3 | 83007859 | NM_133862 |
| Gc |  | group specific component (Gc). | 4.15 | 8.94 | 4.79 | chr5 | 89417511 | NM_008096 |
| Svs3a |  | seminal vesicle secretory protein 3A (Svs3a), transcript variant 1. | 4.67 | 9.18 | 4.51 | chr2 | 164289268 | NM_021363 |
| 9530003J23Rik |  | RIKEN cDNA 9530003J23 gene (9530003J23Rik). | 4.61 | 8.82 | 4.21 | chr10 | 117232237 | NM_029906 |
| Apoa1 | * | apolipoprotein A-I (Apoa1). | 4.47 | 8.66 | 4.19 | chr9 | 46228580 | NM_009692 |
| Pate4 |  | prostate and testis expressed 4 (Pate4). | 4.26 | 8.45 | 4.19 | chr9 | 35607093 | NM_020264 |
| Aldob | * | aldolase B, fructose-bisphosphate (Aldob). | 6.31 | 10.29 | 3.98 | chr4 | 49535995 | NM_144903 |
| Kap |  | kidney androgen regulated protein (Kap). | 4.51 | 8.36 | 3.85 | chr6 | 133849854 | NM_010594 |
| Hpx |  | hemopexin (Hpx). | 4.51 | 8.34 | 3.83 | chr7 | 105591611 | NM_017371 |
| Apob | * | apolipoprotein B (Apob). | 5.15 | 8.96 | 3.81 | chr12 | 7977648 | NM_009693 |
| Serpina1b |  | serine (or cysteine) preptidase inhibitor, clade A, member 1B (Serpina1b). | 4.30 | 8.11 | 3.80 | chr12 | 103728156 | NM_009244 |
| Ahsg | * | alpha-2-HS-glycoprotein (Ahsg), transcript variant 2. | 5.19 | 8.90 | 3.71 | chr16 | 22892015 | NM_001276449 |
| Mug1 |  | murinoglobulin 1 (Mug1). | 5.12 | 8.78 | 3.66 | chr6 | 121838541 | NM_008645 |
| Svs1 |  | seminal vesicle secretory protein 1 (Svs1). | 5.07 | 8.66 | 3.60 | chr6 | 48986861 | NM_172888 |
| Kng1 |  | kininogen 1 (Kng1), transcript variant 1. | 3.74 | 7.04 | 3.30 | chr16 | 23057865 | NM_001102411 |
| Hpd |  | 4-hydroxyphenylpyruvic acid dioxygenase (Hpd). | 5.08 | 8.24 | 3.16 | chr5 | 123171807 | NM_008277 |
| Saa4 |  | serum amyloid A 4 (Saa4). | 4.02 | 7.16 | 3.15 | chr7 | 46727998 | NM_011316 |
| Svs3b |  | seminal vesicle secretory protein 3B (Svs3b). | 5.33 | 8.38 | 3.06 | chr2 | 164254363 | NM_173377 |
| Gpx5 |  | glutathione peroxidase 5 (Gpx5). | 4.53 | 7.44 | 2.91 | chr13 | 21286429 | NM_010343 |
| Ces1c | * | carboxylesterase 1C (Ces1c). | 4.36 | 7.26 | 2.90 | chr8 | 93099015 | NM_007954 |
| Serpina1d |  | serine (or cysteine) peptidase inhibitor, clade A, member 1D (Serpina1d). | 5.55 | 8.40 | 2.85 | chr12 | 103763587 | NM_009246 |
| Pzp |  | pregnancy zone protein (Pzp). | 4.24 | 7.03 | 2.79 | chr6 | 128483567 | NM_007376 |
| Ces3a | * | carboxylesterase 3A (Ces3a), transcript variant 2. | 5.20 | 7.81 | 2.61 | chr8 | 105048599 | NM_001164681 |
| Serpina1e |  | serine (or cysteine) peptidase inhibitor, clade A, member 1E (Serpina1e). | 6.60 | 9.06 | 2.46 | chr12 | 103946931 | NM_009247 |
| Sult2a8 | * | sulfotransferase family 2A, dehydroepiandrosterone (DHEA)-preferring, member 8 (Sult2a8), transcript variant 2. | 4.89 | 7.18 | 2.29 | chr7 | 14410686 | NM_001199306 |
| Apoa2 | * | apolipoprotein A-II (Apoa2), transcript variant 1. | 4.77 | 7.05 | 2.28 | chr1 | 171225054 | NM_013474 |
| Apoa4 | * | apolipoprotein A-IV (Apoa4). | 5.19 | 7.45 | 2.25 | chr9 | 46240696 | NM_007468 |
| Fgb |  | fibrinogen beta chain (Fgb). | 5.40 | 7.60 | 2.20 | chr3 | 83042247 | NM_181849 |
| Slc2a2 | * | solute carrier family 2 (facilitated glucose transporter), member 2 (Slc2a2). | 5.04 | 7.14 | 2.09 | chr3 | 28697903 | NM_031197 |
| Apoh | * | apolipoprotein H (Apoh). | 5.56 | 7.61 | 2.05 | chr11 | 108343354 | NM_013475 |
| Gm17482 |  | predicted gene, 17482 [gene_biotype:protein_coding transcript_biotype:protein_coding] | 6.06 | 8.06 | 2.00 | chr6 | 115227343 | ENSMUST00000166681 |
| Gm6613 |  | PREDICTED: predicted gene 6613 (Gm6613). | 7.00 | 8.72 | 1.72 | chr10 | 84794150 | XM_894484.4 |
| LOC100503047 |  | predicted gene 10264 [gene_biotype:protein_coding transcript_biotype:protein_coding] | 6.09 | 7.81 | 1.72 | chr12 | 88323832 | ENSMUST00000091715 |
| H2-M9 |  | histocompatibility 2, M region locus 9 (H2-M9). | 6.34 | 8.01 | 1.67 | chr17 | 36639285 | NM_008205 |
| Slc9a3 |  | solute carrier family 9 (sodium/hydrogen exchanger), member 3 (Slc9a3). | 6.32 | 7.97 | 1.65 | chr13 | 74121515 | NM_001081060 |
| Zim1 |  | zinc finger, imprinted 1 (Zim1). | 7.21 | 8.86 | 1.64 | chr7 | 6675443 | NM_011769 |
| Anxa8 |  | annexin A8 (Anxa8), transcript variant 2. | 7.29 | 8.81 | 1.52 | chr14 | 34085979 | NM_001281845 |
| Fmo3 | * | flavin containing monooxygenase 3 (Fmo3). | 5.56 | 7.08 | 1.52 | chr1 | 162953800 | NM_008030 |
| Mup4 | * | major urinary protein 4 (Mup4). | 5.72 | 7.23 | 1.50 | chr4 | 59956804 | NM_008648 |
| Fras1 |  | Fraser syndrome 1 homolog (human) (Fras1). | 5.81 | 7.27 | 1.46 | chr5 | 96373955 | NM_175473 |
| Krtap5-1 |  | keratin associated protein 5-1 (Krtap5-1). | 8.12 | 9.56 | 1.44 | chr7 | 142296334 | NM_015808 |
| Trdn |  | triadin (Trdn). | 9.10 | 10.52 | 1.42 | chr10 | 33083483 | NM_029726 |
| Fam180a |  | family with sequence similarity 180, member A (Fam180a). | 7.27 | 8.64 | 1.37 | chr6 | 35312744 | NM_173375 |
| Cyp2d13 |  | cytochrome P450, family 2, subfamily d, polypeptide 13 (Cyp2d13), non-coding RNA. | 8.93 | 10.26 | 1.33 | chr15 | 82636187 | NR_003552 |
| Shroom1 |  | shroom family member 1 (Shroom1), transcript variant 1. | 6.21 | 7.54 | 1.33 | chr11 | 53457205 | NM_027917 |
| Mup20 | * | major urinary protein 20 (Mup20). | 5.84 | 7.17 | 1.32 | chr4 | 62050234 | NM_001012323 |
| Actg2 |  | actin, gamma 2, smooth muscle, enteric (Actg2). | 7.42 | 8.72 | 1.30 | chr6 | 83512905 | NM_009610 |
| Gm10318 |  | predicted gene 10318 (Gm10318). | 6.12 | 7.40 | 1.28 | chr10 | 77852859 | NM_001162944 |
| Hsd17b13 | * | hydroxysteroid (17-beta) dehydrogenase 13 (Hsd17b13), transcript variant 1. | 6.69 | 7.96 | 1.27 | chr5 | 103955440 | NM_001163486 |
| Slc38a5 |  | solute carrier family 38, member 5 (Slc38a5). | 7.23 | 8.50 | 1.27 | chrX | 8271133 | NM_172479 |
| Rtn4rl2 |  | reticulon 4 receptor-like 2 (Rtn4rl2). | 6.20 | 7.46 | 1.26 | chr2 | 84871872 | NM_199223 |
| Nubp1 |  | nucleotide binding protein 1 (Nubp1). | 7.62 | 8.87 | 1.25 | chr16 | 10411938 | NM_011955 |
| Rnase2a |  | ribonuclease, RNase A family, 2A (liver, eosinophil-derived neurotoxin) (Rnase2a). | 11.30 | 12.55 | 1.25 | chr14 | 51255261 | NM_053113 |
| C330021F23Rik |  | RIKEN cDNA C330021F23 gene (C330021F23Rik). | 6.32 | 7.57 | 1.25 | chr8 | 25453869 | uc009lfx.1 |
| Krtap9-1 |  | keratin associated protein 9-1 (Krtap9-1). | 6.63 | 7.88 | 1.25 | chr11 | 99873389 | NM_015741 |
| Krtap17-1 |  | keratin associated protein 17-1 (Krtap17-1). | 8.09 | 9.33 | 1.24 | chr11 | 99993232 | NM_001099774 |
| Gm21860 |  | predicted gene, 21860 [gene_biotype:protein_coding transcript_biotype:protein_coding] | 8.20 | 9.43 | 1.23 | chrY | 90754513 | ENSMUST00000177893 |
| Gm21748 |  | predicted gene, 21748 [gene_biotype:protein_coding transcript_biotype:protein_coding] | 8.20 | 9.43 | 1.23 | chrY | 90838869 | ENSMUST00000179623 |
| C8g |  | complement component 8, gamma polypeptide (C8g), transcript variant 2. | 7.97 | 9.19 | 1.22 | chr2 | 25498650 | NM_001271777 |
| Fgf9 |  | fibroblast growth factor 9 (Fgf9). | 7.34 | 8.56 | 1.22 | chr14 | 58070547 | NM_013518 |
| Mpp4 |  | membrane protein, palmitoylated 4 (MAGUK p55 subfamily member 4) (Mpp4), transcript variant 2. | 5.81 | 7.02 | 1.21 | chr1 | 59120935 | NM_145143 |
| Trp53bp1 |  | transformation related protein 53 binding protein 1 (Trp53bp1), transcript variant 1. | 6.32 | 7.53 | 1.21 | chr2 | 121193263 | NM_013735 |
| Mat1a | * | methionine adenosyltransferase I, alpha (Mat1a). | 5.97 | 7.17 | 1.20 | chr14 | 41105033 | NM_133653 |
| Pemt | * | phosphatidylethanolamine N-methyltransferase (Pemt), transcript variant 2. | 8.77 | 9.97 | 1.19 | chr11 | 59970614 | NM_008819 |
| Eya1 |  | eyes absent 1 homolog (Drosophila) (Eya1), transcript variant 2. | 6.88 | 8.08 | 1.19 | chr1 | 14168954 | NM_001252192 |
| D6Ertd527e |  | DNA segment, Chr 6, ERATO Doi 527, expressed (D6Ertd527e), transcript variant 1. | 6.15 | 7.34 | 1.19 | chr6 | 87104746 | NM_001167937 |
| Dnaja4 | * | DnaJ (Hsp40) homolog, subfamily A, member 4 (Dnaja4). | 9.35 | 10.53 | 1.19 | chr9 | 54698873 | NM_021422 |
| Gsta3 | * | glutathione S-transferase, alpha 3 (Gsta3), transcript variant 1. | 9.96 | 11.14 | 1.18 | chr1 | 21240585 | NM_001077353 |
| Angpt4 | * | angiopoietin 4 (Angpt4). | 5.88 | 7.04 | 1.16 | chr2 | 151911210 | NM_009641 |
| Ndrg2 |  | N-myc downstream regulated gene 2 (Ndrg2), transcript variant 2. | 10.33 | 11.47 | 1.15 | chr14 | 51905271 | NM_001145959 |
| Slc52a3 |  | solute carrier protein family 52, member 3 (Slc52a3), transcript variant 2. | 5.89 | 7.04 | 1.14 | chr2 | 151996511 | NM_001164819 |
| Ccl8 |  | chemokine (C-C motif) ligand 8 (Ccl8). | 9.48 | 10.61 | 1.12 | chr11 | 82115185 | NM_021443 |
| Slc30a3 |  | solute carrier family 30 (zinc transporter), member 3 (Slc30a3). | 6.05 | 7.17 | 1.12 | chr5 | 31086106 | NM_011773 |
| Cdh2 |  | cadherin 2 (Cdh2). | 7.12 | 8.24 | 1.12 | chr18 | 16588877 | NM_007664 |
| Spink1 |  | serine peptidase inhibitor, Kazal type 1 (Spink1). | 6.11 | 7.22 | 1.11 | chr18 | 43728069 | NM_009258 |
| Apoc2 | * | apolipoprotein C-II (Apoc2), transcript variant 1. | 8.29 | 9.40 | 1.11 | chr7 | 19671579 | NM_001277944 |
| F2 |  | coagulation factor II (F2). | 6.32 | 7.42 | 1.10 | chr2 | 91625320 | NM_010168 |
| Arhgef19 | * | Rho guanine nucleotide exchange factor (GEF) 19 (Arhgef19). | 6.02 | 7.12 | 1.10 | chr4 | 141239499 | NM_172520 |
| Cuzd1 |  | CUB and zona pellucida-like domains 1 (Cuzd1). | 7.03 | 8.12 | 1.10 | chr7 | 131308554 | NM_008411 |
| Sprr2j-ps |  | small proline-rich protein 2J, pseudogene (Sprr2j-ps), non-coding RNA. | 6.64 | 7.74 | 1.10 | chr3 | 92418087 | NR_003185 |
| Osbpl6 | * | oxysterol binding protein-like 6 (Osbpl6), transcript variant 1. | 6.67 | 7.77 | 1.09 | chr2 | 76406508 | NM_145525 |
| Vmn1r208 |  | vomeronasal 1 receptor 208 (Vmn1r208). | 6.09 | 7.18 | 1.09 | chr13 | 22772327 | NM_134218 |
| Cd247 |  | CD247 antigen (Cd247), transcript variant zeta. | 6.82 | 7.90 | 1.08 | chr1 | 165781075 | NM_001113391 |
| Klhl17 |  | kelch-like 17 (Klhl17). | 6.59 | 7.67 | 1.07 | chr4 | 156229044 | NM_198305 |
| Prkd1 | * | protein kinase D1 (Prkd1). | 8.35 | 9.42 | 1.07 | chr12 | 50341231 | NM_008858 |
| Loxl4 |  | lysyl oxidase-like 4 (Loxl4), transcript variant 1. | 7.14 | 8.21 | 1.07 | chr19 | 42592279 | NM_001164311 |
| Adcy1 |  | adenylate cyclase 1 (Adcy1). | 6.15 | 7.22 | 1.07 | chr11 | 7063489 | NM_009622 |
| Ass1 |  | argininosuccinate synthetase 1 (Ass1). | 7.14 | 8.20 | 1.07 | chr2 | 31470207 | NM_007494 |
| Fam110c |  | family with sequence similarity 110, member C (Fam110c). | 7.19 | 8.26 | 1.06 | chr12 | 31073968 | NM_027828 |
| Efnb2 |  | ephrin B2 (Efnb2). | 11.79 | 12.85 | 1.06 | chr8 | 8617434 | NM_010111 |
| Krt6a |  | keratin 6A (Krt6a). | 6.27 | 7.33 | 1.06 | chr15 | 101676023 | NM_008476 |
| Mup5 | * | major urinary protein 5 (Mup5). | 6.08 | 7.13 | 1.05 | chr4 | 61831319 | NM_008649 |
| Pdss2 |  | prenyl (solanesyl) diphosphate synthase, subunit 2 (Pdss2), transcript variant 2. | 7.40 | 8.44 | 1.05 | chr10 | 43221486 | NM_001168289 |
| Gm13304 |  | predicted gene 13304 (Gm13304). | 11.90 | 12.94 | 1.04 | chr4 | 42612123 | uc008snn.1 |
| Ccl21b |  | chemokine (C-C motif) ligand 21B (leucine) (Ccl21b). | 11.90 | 12.94 | 1.04 | chr4_GL456350_random | 1 | NM_011335 |
| Ccl21a |  | chemokine (C-C motif) ligand 21A (serine) (Ccl21a). | 11.99 | 13.03 | 1.04 | chr4_JH584294_random | 120655 | NM_011124 |
| Lamc3 |  | laminin gamma 3 (Lamc3). | 7.27 | 8.31 | 1.03 | chr2 | 31887281 | NM_011836 |
| Lgals2 |  | lectin, galactose-binding, soluble 2 (Lgals2). | 6.07 | 7.10 | 1.03 | chr15 | 78850860 | NM_025622 |
| C6 |  | complement component 6 (C6). | 9.10 | 10.13 | 1.03 | chr15 | 4727175 | NM_016704 |
| Gm11111 |  | predicted gene 11111 [gene_biotype:protein_coding transcript_biotype:protein_coding] | 6.94 | 7.97 | 1.03 | chr5 | 98552695 | ENSMUST00000112956 |
| Pkhd1l1 |  | polycystic kidney and hepatic disease 1-like 1 (Pkhd1l1). | 7.49 | 8.52 | 1.03 | chr15 | 44457553 | NM_138674 |
| Tc2n |  | tandem C2 domains, nuclear (Tc2n), transcript variant 2. | 7.26 | 8.27 | 1.01 | chr12 | 101645443 | NM_001082976 |
| Prnd |  | prion protein dublet (Prnd), transcript variant 2. | 7.59 | 8.60 | 1.01 | chr2 | 131909928 | NM_001126338 |
| Gm17530 |  | predicted gene, 17530 | 7.33 | 8.34 | 1.01 | chr9 | 86546682 | ENSMUST00000167814 |

Up-regulated (>2.0-fold, signal>2^7^) genes were identified from a DNA microarray analysis of epididymal fat between WT_A12 and KO_A12 mice fed the high-fat diet for 12 weeks. The number of signal and fold was the base two logarithm. *Known as the glucose or lipid metabolism-related genes. Gray background genes were measured these mRNA levels by real time qPCR.

^a^Fold change was calculated by the gene signal in KO_A12 relative to that in WT_A12 mice.

^b^Chr, chromosome.
