# Supplementary material for: Ablation of Iah1, a candidate gene for diet-induced fatty liver, does not affect liver lipid accumulation in mice
Source: PLoS One. 2020 May 14;15(5):e0233087. doi: 10.1371/journal.pone.0233087 (PMC7224509; doi:10.1371/journal.pone.0233087)
Supplement: S5 Table — (DOCX) [file pone.0233087.s007.docx]

**S5 Table. Downregulated genes in the epididymal fat of A/J-12^SM^ *Iah1*-KO (KO_A12) mice.**

| Gene Symbol | | Gene name | WT: Signal | KO: Signal | Fold change^a^ | Chr^b^ | Start | Gene Accession |
| --- | --- | --- | --- | --- | --- | --- | --- | --- |
| Ucp1 | * | uncoupling protein 1 (mitochondrial, proton carrier) (Ucp1). | 7.47 | 4.78 | -2.69 | chr8 | 83290348 | NM_009463 |
| Prm2 |  | protamine 2 (Prm2). | 8.76 | 6.09 | -2.67 | chr16 | 10791380 | NM_008933 |
| Adam5 |  | a disintegrin and metallopeptidase domain 5 (Adam5), transcript variant 2. | 7.36 | 4.72 | -2.64 | chr8 | 24727093 | NM_001272057 |
| Sfrp4 | * | secreted frizzled-related protein 4 (Sfrp4). | 11.31 | 9.19 | -2.12 | chr13 | 19623175 | NM_016687 |
| Il1rn | * | interleukin 1 receptor antagonist (Il1rn), transcript variant 2. | 11.33 | 9.24 | -2.08 | chr2 | 24336853 | NM_001039701 |
| Foxp2 |  | forkhead box P2 (Foxp2), transcript variant 3. | 7.14 | 5.20 | -1.94 | chr6 | 14901349 | NM_001286607 |
| Smcp |  | sperm mitochondria-associated cysteine-rich protein (Smcp). | 7.25 | 5.36 | -1.89 | chr3 | 92583866 | NM_008574 |
| Gas7 |  | growth arrest specific 7 (Gas7), transcript variant 2. | 10.80 | 8.98 | -1.82 | chr11 | 67455437 | NM_001109657 |
| Cd72 |  | CD72 antigen (Cd72), transcript variant 1. | 10.19 | 8.37 | -1.82 | chr4 | 43446462 | NM_001110320 |
| Aprt |  | adenine phosphoribosyl transferase (Aprt). | 9.14 | 7.34 | -1.80 | chr8 | 122574636 | NM_009698 |
| Adam8 |  | a disintegrin and metallopeptidase domain 8 (Adam8), transcript variant 1. | 11.03 | 9.26 | -1.77 | chr7 | 139978932 | NM_007403 |
| Mfap2 |  | microfibrillar-associated protein 2 (Mfap2), transcript variant 2. | 7.66 | 5.98 | -1.68 | chr4 | 141010418 | NM_001161799 |
| Gm10663 |  | predicted gene 10663 [gene_biotype:protein_coding transcript_biotype:protein_coding] | 9.02 | 7.35 | -1.67 | chr8 | 65075033 | ENSMUST00000098716 |
| Gm3448 |  | predicted gene 3448 (Gm3448). | 9.78 | 8.12 | -1.67 | chr17 | 14995783 | uc008ans.2 |
| Akr1c18 |  | aldo-keto reductase family 1, member C18 (Akr1c18). | 7.31 | 5.66 | -1.65 | chr13 | 4132615 | NM_134066 |
| Lgi4 |  | leucine-rich repeat LGI family, member 4 (Lgi4). | 7.51 | 5.86 | -1.64 | chr7 | 31059342 | NM_144556 |
| Gpnmb | * | glycoprotein (transmembrane) nmb (Gpnmb). | 14.96 | 13.35 | -1.60 | chr6 | 49036518 | NM_053110 |
| Gm3448 |  | predicted gene 3448 (Gm3448). | 9.05 | 7.48 | -1.57 | chr17 | 15027151 | NM_001123367 |
| Itgb2 |  | integrin beta 2 (Itgb2). | 10.47 | 8.92 | -1.55 | chr10 | 77530252 | NM_008404 |
| Mfap5 |  | microfibrillar associated protein 5 (Mfap5). | 13.71 | 12.20 | -1.51 | chr6 | 122505845 | NM_015776 |
| P2ry6 |  | pyrimidinergic receptor P2Y, G-protein coupled, 6 (P2ry6). | 10.73 | 9.23 | -1.51 | chr7 | 100937634 | NM_183168 |
| Itgad |  | integrin, alpha D (Itgad). | 8.13 | 6.65 | -1.48 | chr7 | 128154376 | NM_001029872 |
| Clec7a |  | C-type lectin domain family 7, member a (Clec7a), transcript variant 1, coding. | 12.71 | 11.24 | -1.47 | chr6 | 129461591 | NM_020008 |
| Tnfrsf11a |  | tumor necrosis factor receptor superfamily, member 11a, NFKB activator (Tnfrsf11a). | 8.61 | 7.14 | -1.47 | chr1 | 105780723 | NM_009399 |
| Gm3417 |  | predicted gene 3417 (Gm3417). | 10.16 | 8.72 | -1.45 | chr17 | 14964189 | NM_001123368 |
| Rnf128 |  | ring finger protein 128 (Rnf128), transcript variant 2. | 8.18 | 6.74 | -1.44 | chrX | 139563316 | NM_001254761 |
| St18 |  | suppression of tumorigenicity 18 (St18), transcript variant 1. | 7.90 | 6.47 | -1.44 | chr1 | 6487231 | NM_001244692 |
| Efhd1 |  | EF hand domain containing 1 (Efhd1). | 8.19 | 6.76 | -1.43 | chr1 | 87264363 | NM_028889 |
| Cidea | * | cell death-inducing DNA fragmentation factor, alpha subunit-like effector A (Cidea). | 8.89 | 7.47 | -1.42 | chr18 | 67343564 | NM_007702 |
| Atp2b4 |  | ATPase, Ca++ transporting, plasma membrane 4 [gene_biotype:protein_coding transcript_biotype:protein_coding] | 7.18 | 5.76 | -1.42 | chr1 | 133701639 | ENSMUST00000167348 |
| Pdgfra |  | platelet derived growth factor receptor, alpha polypeptide (Pdgfra), transcript variant 2. | 9.50 | 8.09 | -1.42 | chr5 | 75152291 | NM_001083316 |
| Robo1 |  | roundabout homolog 1 (Drosophila) (Robo1). | 7.03 | 5.62 | -1.41 | chr16 | 72663149 | NM_019413 |
| Pdk4 | * | pyruvate dehydrogenase kinase, isoenzyme 4 (Pdk4). | 13.19 | 11.81 | -1.38 | chr6 | 5483351 | NM_013743 |
| Mfsd12 |  | major facilitator superfamily domain containing 12 (Mfsd12). | 8.19 | 6.83 | -1.36 | chr10 | 81357491 | NM_028657 |
| Cd248 |  | CD248 antigen, endosialin (Cd248). | 12.03 | 10.68 | -1.35 | chr19 | 5068078 | NM_054042 |
| Pla1a | * | phospholipase A1 member A (Pla1a). | 11.38 | 10.03 | -1.35 | chr16 | 38396117 | NM_134102 |
| Lgi2 |  | leucine-rich repeat LGI family, member 2 (Lgi2), transcript variant 1. | 8.73 | 7.38 | -1.35 | chr5 | 52537864 | NM_144945 |
| P2rx4 |  | purinergic receptor P2X, ligand-gated ion channel 4 (P2rx4), transcript variant 1. | 11.61 | 10.27 | -1.34 | chr5 | 122707557 | NM_011026 |
| Gm12250 |  | predicted gene 12250 (Gm12250). | 7.26 | 5.92 | -1.34 | chr11 | 58183843 | NM_001135115 |
| Cass4 |  | Cas scaffolding protein family member 4 (Cass4), transcript variant 2. | 8.46 | 7.13 | -1.33 | chr2 | 172393794 | NM_001080820 |
| Fhl4 |  | four and a half LIM domains 4 (Fhl4). | 7.41 | 6.09 | -1.33 | chr10 | 85097019 | NM_010214 |
| Aar2 |  | AAR2 splicing factor homolog (S. cerevisiae) (Aar2), transcript variant 1. | 7.74 | 6.42 | -1.33 | chr2 | 156547576 | NM_001164818 |
| Dzip1 |  | DAZ interacting protein 1 (Dzip1), transcript variant 1. | 8.21 | 6.89 | -1.32 | chr14 | 118875520 | NM_025943 |
| Nox4 |  | NADPH oxidase 4 (Nox4), transcript variant 2. | 7.52 | 6.20 | -1.32 | chr7 | 87246096 | NM_001285833 |
| Slc37a2 | * | solute carrier family 37 (glycerol-3-phosphate transporter), member 2 (Slc37a2), transcript variant 1. | 10.84 | 9.52 | -1.32 | chr9 | 37229149 | NM_001145960 |
| Atp6v0d2 |  | ATPase, H+ transporting, lysosomal V0 subunit D2 (Atp6v0d2). | 14.26 | 12.95 | -1.32 | chr4 | 19876838 | NM_175406 |
| Rgs2 |  | regulator of G-protein signaling 2 (Rgs2). | 8.79 | 7.48 | -1.31 | chr1 | 143999338 | NM_009061 |
| Lum |  | lumican (Lum). | 15.17 | 13.87 | -1.31 | chr10 | 97565501 | NM_008524 |
| Fbxo5 |  | F-box protein 5 (Fbxo5). | 8.21 | 6.90 | -1.31 | chr10 | 5799158 | NM_025995 |
| Fst |  | follistatin (Fst), transcript variant 2. | 7.74 | 6.43 | -1.31 | chr13 | 114452262 | NM_008046 |
| Cd37 |  | CD37 antigen (Cd37), transcript variant 3. | 12.08 | 10.77 | -1.30 | chr7 | 45233632 | NM_007645 |
| Rpa3 |  | replication protein A3 (Rpa3). | 8.74 | 7.44 | -1.30 | chr6 | 8255936 | NM_026632 |
| LOC100504642 |  | PREDICTED: X-linked lymphocyte-regulated protein PM1-like, transcript variant 1 (LOC100504642). | 7.08 | 5.78 | -1.30 | chrY | 89700321 | XM_003085596.1 |
| Gm4841 |  | predicted gene 4841 (Gm4841). | 7.55 | 6.25 | -1.30 | chr18 | 60268301 | NM_001034859 |
| Ahnak2 |  | AHNAK nucleoprotein 2 (cDNA clone MGC:170093 IMAGE:8861488), complete cds. | 9.14 | 7.85 | -1.29 | chr12 | 112772193 | BC138468 |
| Fyb |  | FYN binding protein (Fyb), transcript variant 2. | 9.50 | 8.22 | -1.28 | chr15 | 6579847 | NM_001278269 |
| Tlr8 |  | toll-like receptor 8 (Tlr8), transcript variant 1. | 10.95 | 9.67 | -1.28 | chrX | 167242696 | NM_133212 |
| Trem2 |  | triggering receptor expressed on myeloid cells 2 (Trem2), transcript variant 2. | 12.00 | 10.72 | -1.27 | chr17 | 48346401 | NM_001272078 |
| Gm7120 |  | predicted gene 7120 (Gm7120), transcript variant 1. | 8.06 | 6.79 | -1.27 | chr13 | 119487941 | NM_001039244 |
| Ms4a14 |  | PREDICTED: membrane-spanning 4-domains, subfamily A, member 14 (Ms4a14). | 9.70 | 8.43 | -1.27 | chr19 | 11301494 | XM_357051.5 |
| Mmp3 | * | matrix metallopeptidase 3 (Mmp3). | 7.05 | 5.79 | -1.26 | chr9 | 7445822 | NM_010809 |
| Panx1 | * | pannexin 1 (Panx1). | 7.02 | 5.76 | -1.26 | chr9 | 15005161 | NM_019482 |
| Nat10 |  | N-acetyltransferase 10 (Nat10). | 9.24 | 7.98 | -1.25 | chr2 | 103721256 | NM_153126 |
| Rab43 |  | RAB43, member RAS oncogene family (Rab43), transcript variant 1. | 7.94 | 6.69 | -1.25 | chr6 | 87788853 | NM_001039394 |
| Egr2 | * | early growth response 2 (Egr2). | 10.32 | 9.08 | -1.25 | chr10 | 67535475 | NM_010118 |
| Acer3 | * | alkaline ceramidase 3 (Acer3). | 10.36 | 9.12 | -1.24 | chr7 | 98211987 | NM_025408 |
| Ncf2 |  | neutrophil cytosolic factor 2 (Ncf2). | 10.80 | 9.57 | -1.23 | chr1 | 152807257 | NM_010877 |
| Cpa3 |  | carboxypeptidase A3, mast cell (Cpa3). | 11.66 | 10.43 | -1.23 | chr3 | 20215616 | NM_007753 |
| Fgf18 |  | fibroblast growth factor 18 (Fgf18), transcript variant 1. | 9.45 | 8.23 | -1.22 | chr11 | 33116978 | NM_008005 |
| Tyrobp |  | TYRO protein tyrosine kinase binding protein (Tyrobp). | 10.80 | 9.58 | -1.22 | chr7 | 30413788 | NM_011662 |
| Tlr13 |  | toll-like receptor 13 (Tlr13). | 10.84 | 9.62 | -1.22 | chrX | 106143204 | NM_205820 |
| Klf11 |  | Kruppel-like factor 11 (Klf11). | 9.21 | 7.99 | -1.22 | chr12 | 24651371 | NM_178357 |
| Glipr1 |  | GLI pathogenesis-related 1 (glioma) (Glipr1). | 11.16 | 9.94 | -1.22 | chr10 | 111985448 | NM_028608 |
| Plekho2 |  | pleckstrin homology domain containing, family O member 2 (Plekho2). | 11.52 | 10.31 | -1.21 | chr9 | 65552577 | NM_153119 |
| Dbnl |  | drebrin-like (Dbnl), transcript variant 1. | 8.38 | 7.17 | -1.21 | chr11 | 5788483 | NM_001146308 |
| Dhrs9 |  | dehydrogenase/reductase (SDR family) member 9 (Dhrs9). | 7.74 | 6.53 | -1.21 | chr2 | 69380445 | NM_175512 |
| Crisp2 |  | cysteine-rich secretory protein 2 (Crisp2), transcript variant 2. | 7.28 | 6.08 | -1.21 | chr17 | 40764734 | NM_001204071 |
| Gm4951 |  | predicted gene 4951 (Gm4951). | 8.70 | 7.50 | -1.20 | chr18 | 60212077 | NM_001033767 |
| Nceh1 | * | neutral cholesterol ester hydrolase 1 (Nceh1). | 13.49 | 12.29 | -1.20 | chr3 | 27182965 | NM_178772 |
| Metrnl | * | meteorin, glial cell differentiation regulator-like (Metrnl). | 10.97 | 9.78 | -1.20 | chr11 | 121681044 | NM_144797 |
| Gas2l3 |  | growth arrest-specific 2 like 3 (Gas2l3), transcript variant 1. | 10.70 | 9.50 | -1.20 | chr10 | 89408540 | NM_001033331 |
| Ankrd63 |  | ankyrin repeat domain 63 (Ankrd63). | 7.14 | 5.95 | -1.19 | chr2 | 118699103 | NM_001081971 |
| Cd200r4 |  | CD200 receptor 4 (Cd200r4). | 7.29 | 6.11 | -1.18 | chr16 | 44811733 | NM_207244 |
| Fermt3 |  | fermitin family homolog 3 (Drosophila) (Fermt3). | 8.43 | 7.25 | -1.18 | chr19 | 6998958 | NM_153795 |
| Clec12a |  | C-type lectin domain family 12, member a (Clec12a). | 13.35 | 12.17 | -1.18 | chr6 | 129342691 | NM_177686 |
| Atf3 | * | activating transcription factor 3 (Atf3). | 10.96 | 9.78 | -1.17 | chr1 | 191170296 | NM_007498 |
| 2700049A03Rik |  | RIKEN cDNA 2700049A03 gene (2700049A03Rik), transcript variant 1. | 7.41 | 6.24 | -1.17 | chr12 | 71136848 | NM_001163378 |
| Gm5431 |  | predicted gene 5431 (Gm5431). | 9.42 | 8.25 | -1.17 | chr11 | 48887422 | NM_001024230 |
| Iigp1 |  | interferon inducible GTPase 1 (Iigp1), transcript variant 2. | 9.49 | 8.32 | -1.17 | chr18 | 60376028 | NM_001146275 |
| Iah1 | * | isoamyl acetate-hydrolyzing esterase 1 homolog (S. cerevisiae) (Iah1). | 9.52 | 8.35 | -1.17 | chr12 | 21316389 | NM_026347 |
| Rnf180 |  | ring finger protein 180 (Rnf180). | 8.11 | 6.94 | -1.17 | chr13 | 105130575 | NM_027934 |
| Cspg4 |  | chondroitin sulfate proteoglycan 4 (Cspg4). | 9.06 | 7.90 | -1.16 | chr9 | 56865104 | NM_139001 |
| Prkcb | * | protein kinase C, beta (Prkcb), transcript variant 1. | 9.11 | 7.95 | -1.16 | chr7 | 122288751 | NM_008855 |
| Gabra3 |  | gamma-aminobutyric acid (GABA) A receptor, subunit alpha 3 (Gabra3). | 9.51 | 8.35 | -1.16 | chrX | 72432676 | NM_008067 |
| Slc11a1 |  | solute carrier family 11 (proton-coupled divalent metal ion transporters), member 1 (Slc11a1). | 12.67 | 11.51 | -1.16 | chr1 | 74375203 | NM_013612 |
| Scpep1 |  | serine carboxypeptidase 1 (Scpep1). | 12.52 | 11.37 | -1.15 | chr11 | 88924020 | NM_029023 |
| Ppp1r16a |  | protein phosphatase 1, regulatory (inhibitor) subunit 16A (Ppp1r16a). | 7.13 | 5.97 | -1.15 | chr15 | 76671615 | NM_033371 |
| Irgm1 | * | immunity-related GTPase family M member 1 (Irgm1). | 10.54 | 9.39 | -1.15 | chr11 | 48861968 | NM_008326 |
| Lrrc17 |  | leucine rich repeat containing 17 (Lrrc17). | 8.59 | 7.44 | -1.15 | chr5 | 21543527 | NM_028977 |
| Slfn2 |  | schlafen 2 (Slfn2). | 9.99 | 8.85 | -1.14 | chr11 | 83065112 | NM_011408 |
| Creb5 | * | cAMP responsive element binding protein 5 (Creb5). | 9.64 | 8.50 | -1.14 | chr6 | 53287295 | NM_172728 |
| Rassf4 |  | Ras association (RalGDS/AF-6) domain family member 4 (Rassf4). | 10.87 | 9.73 | -1.14 | chr6 | 116633008 | NM_178045 |
| Dennd4b |  | DENN/MADD domain containing 4B (Dennd4b). | 9.84 | 8.70 | -1.14 | chr3 | 90265185 | NM_201407 |
| Pianp |  | PILR alpha associated neural protein (Pianp), transcript variant 2. | 11.49 | 10.36 | -1.13 | chr6 | 124996694 | NM_001145926 |
| Cdc5l |  | cell division cycle 5-like (S. pombe) (Cdc5l). | 7.44 | 6.31 | -1.13 | chr17 | 45391887 | NM_152810 |
| Cd14 |  | CD14 antigen (Cd14). | 9.72 | 8.59 | -1.13 | chr18 | 36725067 | NM_009841 |
| Cd80 |  | CD80 antigen (Cd80). | 9.23 | 8.11 | -1.12 | chr16 | 38455561 | NM_009855 |
| Slc25a45 |  | solute carrier family 25, member 45 (Slc25a45). | 8.41 | 7.28 | -1.12 | chr19 | 5877808 | NM_134154 |
| Pik3r5 |  | phosphoinositide-3-kinase, regulatory subunit 5, p101 (Pik3r5). | 9.89 | 8.77 | -1.12 | chr11 | 68432121 | NM_177320 |
| Pira6 |  | paired-Ig-like receptor A6 (Pira6), transcript variant 1. | 10.45 | 9.32 | -1.12 | chr7 | 4274143 | NM_008848 |
| Lgals3 |  | lectin, galactose binding, soluble 3 (Lgals3), transcript variant 1. | 13.21 | 12.09 | -1.12 | chr14 | 47367751 | NM_001145953 |
| Naip5 |  | NLR family, apoptosis inhibitory protein 5 (Naip5). | 9.09 | 7.97 | -1.12 | chr13 | 100211739 | NM_010870 |
| Dok1 |  | docking protein 1 (Dok1), transcript variant 1. | 7.19 | 6.08 | -1.12 | chr6 | 83030934 | NM_010070 |
| Tnfrsf1b |  | tumor necrosis factor receptor superfamily, member 1b (Tnfrsf1b). | 11.30 | 10.19 | -1.11 | chr4 | 145212368 | NM_011610 |
| Pigv |  | phosphatidylinositol glycan anchor biosynthesis, class V (Pigv), transcript variant 2. | 7.17 | 6.06 | -1.11 | chr4 | 133660387 | NM_001145955 |
| Ckb |  | creatine kinase, brain (Ckb). | 12.37 | 11.26 | -1.11 | chr12 | 111669355 | NM_021273 |
| Anpep |  | alanyl (membrane) aminopeptidase (Anpep). | 15.04 | 13.94 | -1.11 | chr7 | 79821803 | NM_008486 |
| Galnt7 |  | UDP-N-acetyl-alpha-D-galactosamine: polypeptide N-acetylgalactosaminyltransferase 7 (Galnt7), transcript variant 2. | 8.61 | 7.51 | -1.10 | chr8 | 57523825 | NM_001167981 |
| Ackr3 | * | atypical chemokine receptor 3 (Ackr3), transcript variant 1. | 10.96 | 9.86 | -1.10 | chr1 | 90203980 | NM_001271607 |
| Bcl2a1d |  | B cell leukemia/lymphoma 2 related protein A1d (Bcl2a1d). | 8.81 | 7.71 | -1.10 | chr9 | 88723287 | NM_007536 |
| Stap1 |  | signal transducing adaptor family member 1 (Stap1), transcript variant 1. | 8.20 | 7.11 | -1.09 | chr5 | 86071746 | NM_019992 |
| Lair1 |  | leukocyte-associated Ig-like receptor 1 (Lair1), transcript variant a. | 8.96 | 7.88 | -1.09 | chr7 | 4003288 | NM_001113474 |
| Zfp125 |  | zinc finger protein 125 [gene_biotype:protein_coding transcript_biotype:protein_coding] | 7.32 | 6.24 | -1.08 | chr12 | 20899822 | ENSMUST00000079237 |
| Lrrc27 |  | leucine rich repeat containing 27 (Lrrc27), transcript variant 2. | 11.50 | 10.42 | -1.08 | chr7 | 139212988 | NM_001143755 |
| Tbxas1 |  | thromboxane A synthase 1, platelet (Tbxas1). | 11.73 | 10.65 | -1.08 | chr6 | 38875404 | NM_011539 |
| Ptprs |  | protein tyrosine phosphatase, receptor type, S (Ptprs), transcript variant 2. | 12.66 | 11.58 | -1.08 | chr17 | 56412426 | NM_001252453 |
| Gm12689 |  | predicted gene 12689 [gene_biotype:protein_coding transcript_biotype:protein_coding] | 7.24 | 6.16 | -1.08 | chr4 | 99295900 | ENSMUST00000094955 |
| Dpep2 |  | dipeptidase 2 (Dpep2), transcript variant 3. | 11.19 | 10.12 | -1.08 | chr8 | 105984944 | NM_176913 |
| Ccdc88a |  | coiled coil domain containing 88A (Ccdc88a). | 9.77 | 8.70 | -1.08 | chr11 | 29373658 | NM_176841 |
| Slc38a1 |  | solute carrier family 38, member 1 (Slc38a1), transcript variant 2. | 9.44 | 8.37 | -1.08 | chr15 | 96571418 | NM_001166456 |
| Tmem67 |  | transmembrane protein 67 (Tmem67), transcript variant 1. | 7.85 | 6.77 | -1.07 | chr4 | 12039355 | NM_177861 |
| Rps6kc1 |  | ribosomal protein S6 kinase polypeptide 1 (Rps6kc1). | 7.03 | 5.96 | -1.07 | chr1 | 190700202 | NM_178775 |
| Fbn1 | * | fibrillin 1 (Fbn1). | 14.39 | 13.32 | -1.07 | chr2 | 125300594 | NM_007993 |
| Ankdd1a |  | ankyrin repeat and death domain containing 1A [gene_biotype:protein_coding transcript_biotype:protein_coding] | 8.15 | 7.08 | -1.07 | chr9 | 65488470 | ENSMUST00000061766 |
| Ssc5d |  | scavenger receptor cysteine rich family, 5 domains (Ssc5d). | 7.17 | 6.10 | -1.06 | chr7 | 4925844 | NM_173008 |
| F7 |  | coagulation factor VII (F7). | 7.79 | 6.73 | -1.06 | chr8 | 13026034 | NM_010172 |
| Igtp |  | interferon gamma induced GTPase (Igtp). | 8.00 | 6.94 | -1.06 | chr11 | 58199556 | NM_018738 |
| Ecm1 |  | extracellular matrix protein 1 (Ecm1), transcript variant 2. | 13.11 | 12.06 | -1.06 | chr3 | 95734147 | NM_001252653 |
| Lsp1 |  | lymphocyte specific 1 (Lsp1), transcript variant 1. | 11.99 | 10.93 | -1.06 | chr7 | 142460809 | NM_001136071 |
| Map6 |  | microtubule-associated protein 6 (Map6), transcript variant 3. | 9.07 | 8.01 | -1.05 | chr7 | 99267447 | NM_001043355 |
| Ptafr | * | platelet-activating factor receptor (Ptafr). | 13.30 | 12.25 | -1.05 | chr4 | 132564067 | NM_001081211 |
| Ptchd1 |  | patched domain containing 1 (Ptchd1). | 8.24 | 7.18 | -1.05 | chrX | 155569736 | NM_001093750 |
| Man2b2 | * | mannosidase 2, alpha B2 (Man2b2). | 10.61 | 9.56 | -1.05 | chr5 | 36806813 | NM_008550 |
| Prr13 |  | proline rich 13 (Prr13), transcript variant 2. | 12.51 | 11.46 | -1.05 | chr15 | 102459028 | NM_001170911 |
| Map4k1 |  | mitogen-activated protein kinase kinase kinase kinase 1 (Map4k1). | 7.62 | 6.57 | -1.05 | chr7 | 28982050 | NM_008279 |
| Renbp |  | renin binding protein (Renbp), transcript variant 2. | 8.28 | 7.24 | -1.05 | chrX | 73922121 | NM_001164704 |
| B4galt5 | * | UDP-Gal:betaGlcNAc beta 1,4-galactosyltransferase, polypeptide 5 (B4galt5). | 9.91 | 8.86 | -1.04 | chr2 | 167298444 | NM_019835 |
| Dnm1 |  | dynamin 1 (Dnm1), transcript variant 1. | 7.45 | 6.41 | -1.04 | chr2 | 32308471 | NM_010065 |
| Spata33 |  | spermatogenesis associated 33 (Spata33). | 8.12 | 7.08 | -1.04 | chr8 | 123212858 | NM_177279 |
| Rmdn3 |  | regulator of microtubule dynamics 3 (Rmdn3). | 8.93 | 7.89 | -1.04 | chr2 | 119136998 | NM_001033136 |
| Fn1 |  | fibronectin 1 (Fn1), transcript variant 2. | 15.94 | 14.91 | -1.03 | chr1 | 71585473 | NM_001276408 |
| Slc9a3r1 |  | solute carrier family 9 (sodium/hydrogen exchanger), member 3 regulator 1 (Slc9a3r1). | 8.45 | 7.42 | -1.03 | chr11 | 115163341 | NM_012030 |
| Tmem100 |  | transmembrane protein 100 (Tmem100). | 9.86 | 8.83 | -1.03 | chr11 | 90030348 | NM_026433 |
| Syk |  | spleen tyrosine kinase (Syk), transcript variant 2. | 10.74 | 9.71 | -1.03 | chr13 | 52583173 | NM_001198977 |
| Rgs1 |  | regulator of G-protein signaling 1 (Rgs1). | 10.58 | 9.56 | -1.02 | chr1 | 144243972 | NM_015811 |
| Gm8909 |  | predicted gene 8909 (Gm8909). | 10.32 | 9.30 | -1.02 | chr17 | 36164443 | NM_001081032 |
| Lipa | * | lysosomal acid lipase A (Lipa), transcript variant 2. | 14.23 | 13.21 | -1.02 | chr19 | 34492316 | NM_001111100 |
| Sun2 |  | Sad1 and UNC84 domain containing 2 (Sun2), transcript variant 1. | 8.30 | 7.28 | -1.02 | chr15 | 79724068 | NM_001205345 |
| Fcer1g |  | Fc receptor, IgE, high affinity I, gamma polypeptide (Fcer1g). | 14.53 | 13.52 | -1.02 | chr1 | 171229572 | NM_010185 |
| Prrx1 | * | paired related homeobox 1 (Prrx1), transcript variant 3. | 13.72 | 12.70 | -1.02 | chr1 | 163245116 | NM_001025570 |
| Il7r |  | interleukin 7 receptor (Il7r). | 11.87 | 10.86 | -1.01 | chr15 | 9506159 | NM_008372 |
| Sufu |  | suppressor of fused homolog (Drosophila) (Sufu), transcript variant 2. | 9.21 | 8.20 | -1.01 | chr19 | 46396896 | NM_001025391 |
| Ank |  | progressive ankylosis (Ank). | 12.07 | 11.06 | -1.01 | chr15 | 27466677 | NM_020332 |
| Sh3bgrl3 |  | SH3 domain binding glutamic acid-rich protein-like 3 (Sh3bgrl3). | 9.90 | 8.89 | -1.00 | chr4 | 134127406 | NM_080559 |
| Ankrd13b |  | ankyrin repeat domain 13b (Ankrd13b). | 8.34 | 7.33 | -1.00 | chr11 | 77470485 | NM_172945 |
| Pi16 |  | peptidase inhibitor 16 (Pi16). | 7.05 | 6.04 | -1.00 | chr17 | 29318877 | NM_023734 |
| Nans |  | N-acetylneuraminic acid synthase (sialic acid synthase) (Nans). | 9.26 | 8.26 | -1.00 | chr4 | 46489248 | NM_053179 |

Down-regulated (>0.5-fold, signal>2^7^) genes were identified from a DNA microarray analysis of epididymal fat between WT_A12 and KO_A12 mice fed the high-fat diet for 12 weeks. The number of signal and fold was the base two logarithm. *Known as the glucose or lipid metabolism-related genes. Gray background genes were measured these mRNA levels by real time qPCR.

^a^Fold change was calculated by the gene signal in KO_A12 relative to that in WT_A12 mice.

^b^Chr, chromosome.
