# Supplementary material for: Ablation of Iah1, a candidate gene for diet-induced fatty liver, does not affect liver lipid accumulation in mice
Source: PLoS One. 2020 May 14;15(5):e0233087. doi: 10.1371/journal.pone.0233087 (PMC7224509; doi:10.1371/journal.pone.0233087)
Supplement: S1 Raw images — (PDF) [file pone.0233087.s008.pdf]

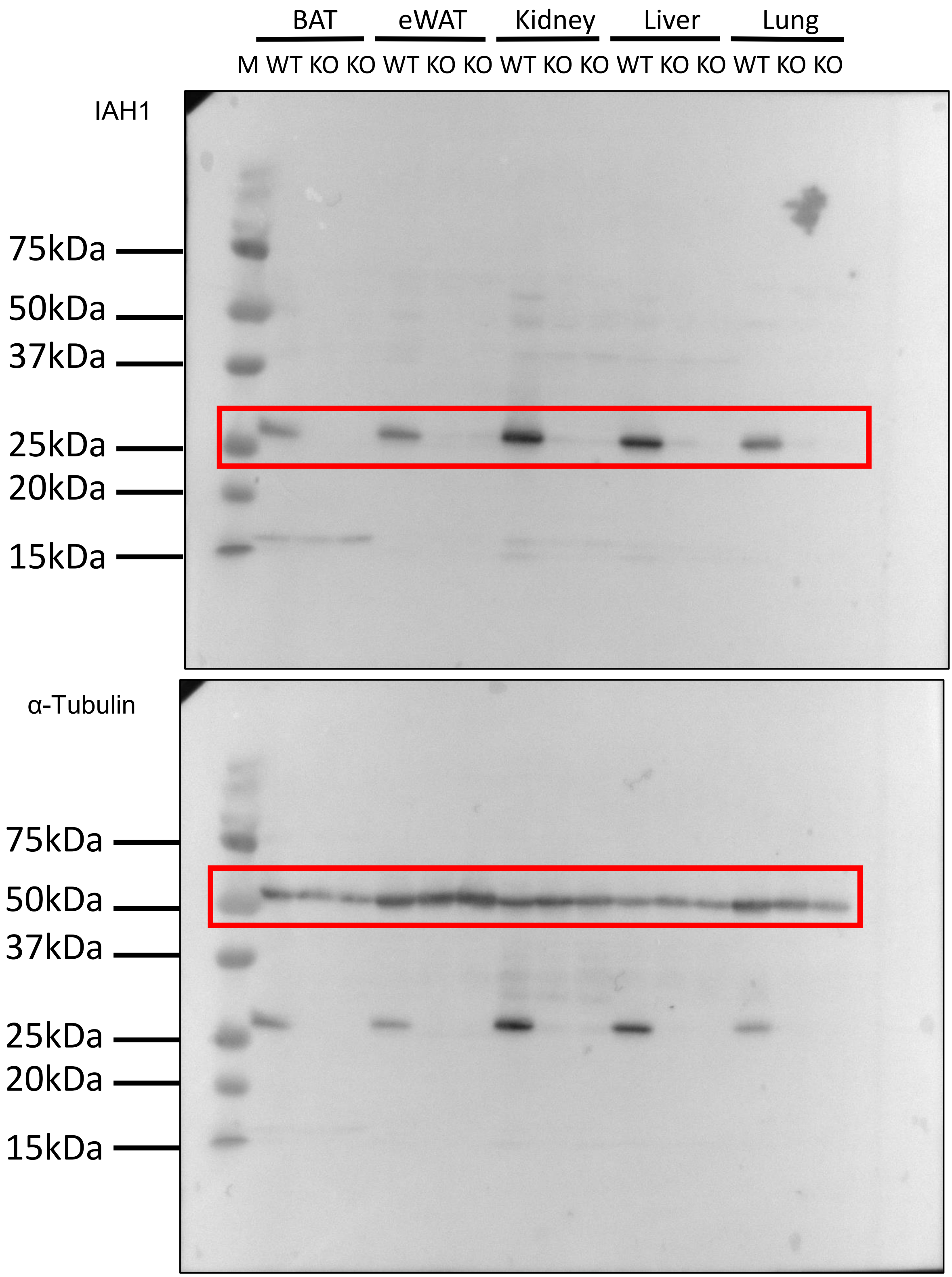

BAT
eWAT
Kidney
Liver
Lung
M WT KO KO WT KO KO WT KO KO WT KO KO WT KO KO
IAH1
75kDa
50kDa
37kDa
25kDa
20kDa
15kDa
α-Tubulin
75kDa
50kDa
37kDa
25kDa
20kDa
15kDa
